# BERT-spaCy hybrid NLP and blockchain-enhanced adaptive CTI for IOC extraction and threat prediction

**DOI:** 10.1038/s41598-025-34505-2

**Published:** 2026-03-02

**Authors:** Shailendra Mishra, Ruba Ahmed Alfahidah, Fayez Alharbi

**Affiliations:** 1 Department of Computer Engineering, College of Computer and Information Sciences, Majmaah University, 11952 Al Majmaah, Saudi Arabia; 2 Department of Information Technology, College of Computer and Information Sciences, Majmaah University, 11952 Al Majmaah, Saudi Arabia

**Keywords:** Cyber threat intelligence, BERT, Blockchain, NLP, Machine learning, Robust inference, Immutable ledger, IOC extraction, Adaptive learning, Engineering, Mathematics and computing

## Abstract

Cyber-attacks pose a significant risk to digital infrastructure, resulting in losses at both individual and organizational levels, underscoring the need for proactive and intelligent defense mechanisms. This study proposes a hybrid Cyber Threat Intelligence (CTI) system integrating an immutable blockchain ledger, adaptive machine-learning models, and natural-language processing algorithms for timely detection, classification, and secure sharing of threat data. The system forecasts future attacks by analyzing aggregated data and recommending mitigation strategies. A BERT-based model, combined with spaCy and regular expressions for extracting Indicators of Compromise (IOCs) from unstructured data, achieved 95% accuracy and a 95.7% F1-score, with a 55% latency reduction (from 120ms to 54ms for 200 reports). Validation used 10-fold cross-validation with paired t-tests across 10,000 Monte Carlo simulations (t = 3.45, *p* < 0.001, Cohen’s d ranging 0.76–1.12 from heatmaps) on CIC-IDS2017 and UNSW-NB15 datasets. The Cross-Dataset Robustness Index (CRI) confirmed strong generalization, with BERT at 0.999, slightly outperforming LSTM (0.998), SVM (0.95), and Naïve Bayes (0.92). The system excels in high-volume data processing, event correlation, and threat detection/response rates. This scalable solution suits Security Operations Centers (SOCs), IoT environments, and financial cybersecurity, providing robust unstructured data handling and adaptability to evolving threats.

## Introduction

 Cybersecurity is vital for ensuring the integrity and reliability of electronic systems that underpin modern life, including finance, healthcare, communication, and industry. However, cyber-attacks are becoming more frequent, complex, and difficult to predict, exploiting system vulnerabilities, human errors, and emerging technologies, thereby compromising business processes and society as a whole^1–3^. These challenges necessitate defensive systems that can learn, adapt, and respond rapidly to evolving threats.

CTI platforms are widely used for detecting, analyzing, and responding to cyber threats. However, traditional CTI solutions face significant obstacles: they struggle with large volumes of unstructured data, real-time identification of new threats, and secure data sharing across organizational boundaries^4–9^. Extracting useful IOCs from sources like emails, social media, or incident reports remains labor-intensive^10,11^. Additionally, without tamper-proof sharing mechanisms, inter-organizational trust may be lacking, hindering collaborative responses and reducing overall cybersecurity efficiency^12^.

To address these limitations, intelligent, adaptive, and secure real-time threat data processing frameworks are essential^13^. Current systems falter in learning about emerging risks due to inefficiencies in handling massive unstructured data streams, particularly in IIoT networks. Modern cyberattacks are dynamic, incorporating malware, DDoS, and advanced persistent threats that exploit system and human vulnerabilities^1,2^. Although CTI systems aim to mitigate them, traditional approaches are limited by their inability to process large unstructured data in real time, adapt to novel attacks, or ensure secure collaboration^4,5^. For example, static models like MITRE ATT&CK offer guidelines but lack flexibility against constantly evolving threats^13^.

Recent studies emphasize enhancing CTI with predictive analytics, machine learning, and distributed intelligence. However, modern systems still exhibit latency in threat detection and cross-organizational trust issues^10,14^. There is an urgent need for structures that continuously learn, evolve, and share actionable threat intelligence securely. Predictive analysis, deep learning, and distributed machine learning are key to addressing these, enabling assessment of vast data, discovery of unseen trends, and rapid response to emerging threats. Governments, businesses, and individuals face risks from cyberattacks, and traditional systems often fail to detect novel ones. A predictive system using ML is essential for recognizing and halting future attacks^15^.

This study provides a foundation for a CTI strategy to preempt cyber threats and enhance cybersecurity. We present a unified CTI model incorporating NLP, adaptive ML, and a blockchain-based ledger. Key focuses include: (1) processing heterogeneous unstructured threat data via a hybrid BERT-enhanced NLP with regular expressions for IOC extraction; (2) enabling adaptive learning for emergent threats; (3) using a lightweight blockchain-inspired immutable ledger for traceable intelligence sharing; (4) ensuring secure, transparent dissemination across organizations; and (5) empirical evaluation of accuracy, F1-score, precision, and recall on benchmark datasets for performance and scalability.

**Research questions**

- RQ1: How can hybrid NLP-ML techniques reliably extract and classify IOCs and attack patterns from unstructured CTI reports, overcoming limitations of static indicators?
- RQ2: How can blockchain-integrated ledgers ensure tamper-proof, traceable CTI sharing across organizations while addressing trust, privacy, and interoperability challenges?
- RQ3: How can confidence-weighted ensembles and adaptive ML mitigate class imbalance in cybersecurity datasets to improve threat prediction accuracy and generalization?

The proposed framework uses adaptive learning to boost detection accuracy and reduce response times. Experiments show accuracy increasing by 18% and SOC response times reduced by 55% (120ms to 54ms), supporting real-world applications in finance, healthcare, and IoT. It enables accelerated threat detection, cross-sector responses, and resilience against diverse cyber-attacks through predictive analysis, adaptive learning, and secure sharing.

The contribution involves the interdependent collaboration between agency representatives and cybersecurity personnel to guarantee efficient and effective cybersecurity measures. The proposed system provides a scalable and decipherable CTI framework that improves the threat detection, classification and protected information exchange. Whereas adaptive ML has a high classification performance.The ledger is blockchain-inspired, providing tamper-proof and transparent collaboration, which reduces risks associated with centralized storage. Experimental results shows that it has greatly improved detection (75 to 93), Table 1 shows 75% → 93% over 12 months and response latency (120 to 54), as compared to that of the previous studies^3,6^, and^16^.

The framework builds upon prior research by providing a confidence-weighted ensemble to make real time corrections, scoring F1 of 95.7% and almost optimal accuracy (95% on IPs, 92% on domains) at IOC extraction. Single-point-of-failures are removed through decentralization and insider threat through unchangeable ledger blocks.

Cross-Dataset Robustness Index (CRI) revealed that BERT performed the best compared to LSTM, SVM, and Naïve Bayes, achieving the highest value of generalization at 0.999. Effect-size visualization using Cohen’s d heat maps (Fig. 7) revealed why class-wise variations in F1 score correlate across datasets, exposing functional disparities among other models. This evaluation recontextualizes BERT’s performance, not only as an exceptionally high-performing model on training data, but as one demonstrating transferable, persistent robustness across disparate network settings. It also points to the intrinsic instability of standard models, which, while better in isolated tests, become unstable under diverse or unpredictable traffic patterns.

The rest of this report is structured as follows. The section “Literature review” provides a literature review about CTI and blockchain, NLP and ML integration. The section “Research methodology” explains the architecture of the framework and the choice of the methodology used. The section "Model performance and analysis" gives experimental procedures, results and subsequent analysis of the same. Lastly, in the section "Conclusions", the paper is completely closed by remarks and recommendations on future research.

**Table 1:** Confusion Matrices.

Model	TP	FN	FP	TN	Accuracy Trend	Observations
BERT	8742	59	42	958	Highest accuracy and balance	Best at detecting both classes with low error rate.
LSTM	8650	151	134	866	High accuracy, balanced performance	Handles both classes better; ideal for real-time detection.
SVM	8604	197	180	820	Moderate accuracy, high FP	Overpredicts DoS as BENIGN; moderate recall but low precision.
Naïve Bayes	8350	451	385	615	High FN & FP	Fails to distinguish BENIGN and DoS due to data imbalance.

## Literature review

The aim of this section will be to look at the research that questions threats and improves cybersecurity by use of modern technology. They are the gathering and evaluation of intelligence information, blockchain technology, and artificial intelligence. Also, many of the CTI linked topics were discussed, including deep learning, blockchain technology, artificial intelligence, and the use of the dark web and hacker forums. This was done as research to fill the gaps that were identified in the past. To present the CTI, the literature review focuses on blending the blockchain technology with the concept of natural language processing. The main focus of the given study is natural language processing, blockchains, and the latest cybersecurity frameworks, as well as the areas that still need further investigation. Following the thematic organization, the literature review was separated into four major sections, namely (1) Existing CTI Frameworks, (2) Applications of NLP in Cybersecurity, and (3) Blockchain in CTI. Coupled with a large base of citations, this systematic procedure reminds of the multidisciplinary nature of our theory and clearly identifies the gap in research where automation, intelligence extraction, and secure exchange of threat information come together.

### Existing cyber threat intelligence (CTI) frameworks

Traditional CTI models, such as the MITRE ATT&CK framework and the Cyber Kill Chain developed by Lockheed Martin, offer algorithmic frameworks to comprehend the processes of updating malware and aggressor behavior^17^. The MITRE ATT&CK framework^15^, provides extensive body of knowledge about adversary techniques and tactics which has been used to establish an analysis of Business Email Compromise (BEC)^18^, and in developing Advanced Persistent Threat (APT) training environments. The Diamond Model of Intrusion Analysis also brings attention to the relationship between the adversary, capabilities, infrastructure, and one of the victims^19^. As the demand for smarter systems that can incorporate AI driven insights and blockchain-secured intelligence sharing increases, so does the need for adaptable, real-time CTI.

The rules of CTI have recently been rewritten using distributed ledger, machine learning, and deep learning technologies^7^. The horizon of CTI is quickly growing, from leveraging heterogeneous information networks to forecast the movements of Advanced Persistent Threats (APTs) to detecting RDoS attacks on satellite-linked healthcare systems with astounding precision. Superior vulnerability detection has been shown by deep learning models such as Bi-LSTM and function-as-sentence approaches, while blockchain-based frameworks offer safe, motivated cross-organizational cooperation. These developments portend a day when CTI will be self-learning, predictive, and completely integrated into the digital fabric of contemporary infrastructure. A threat intelligence system designed to protect smart satellite-based healthcare networks (SmartSat-IIoHT) against cyberattacks that take advantage of the CoAP protocol, a crucial communication standard for medical devices^20^.

The proposed solution performed well, detecting RDoS-CoAP threats with an attack detection accuracy of 97.43%. A strategy for predicting APT group operations using improved CTI was presented in^17^. To increase threat prediction accuracy, the suggested method uses a structured, multi-stage process that includes data collection, enrichment, and connection mapping. A method for developing CTI for vulnerabilities in closed-source software that relies on deep learning was introduced in^21^. LSTM, MLP, OneDNN, and Bi-LSTM are some of the advanced algorithms that the framework uses to accurately identify software vulnerabilities.

Current CTI frameworks have made good progress in specific areas, but they often fall short when it comes to building a complete system. Many studies show strong results in isolated tasks—for example, one approach that treated functions as sentences reached an F1-score of 82.4% and AUC of 93.0%, beating nearly 200 other methods. The same work also found that 60% of vulnerabilities appear first on hacker forums before official reports, making these forums valuable early-warning sources. Several researchers have proposed blockchain-based sharing models to protect privacy and build trust among organizations^3^.

To encourage users to generate, consume, and assess CTI data, the theory uses two incentive systems: monetary rewards and reputation-based bonuses. Using IPFS for off-chain storage, the Hyperledger Fabric-based approach enables the safe and cooperative sharing of threat intelligence. Although problems with data quality, scalability, automation, and privacy persist, it underlines the need of CTI technology in stopping cyber threats. Integration of blockchain and artificial intelligence (AI) technology is now being investigated to solve these problems through improved cybersecurity, simplification of 208 data structures, and development of useful applications. The XGBoost algorithm is used. The same authors’ study of the black web as a CTI source brought to light the trade-off between the scalability problems of automated methods and the manual efficiency on small datasets. Dekker M et al.^22^ developed CyberEntRel, a deep learning-based model intended to extract cyber entities and their interactions, in order to push the boundaries of CTI and provide real-time insights for preventing complex attacks.

Additionally^23^, presented a unique architecture for email CTI sharing that detects spam and dangers in encrypted communications using metadata and ontologies rather than message content. Recent studies by^3,7,14^, have been emphasize the growing necessity of adaptive response tactics, automation, and trust in contemporary threat intelligence systems. The system’s architecture change toward collaborative and real-time intelligence sharing is directly influenced by these investigations. They are applicable in the identification of tactics, techniques, and procedures (TTPs) but their rigid and rule form restricts flexibility to the dynamic and changing threats of the current era. As a result, studies have advanced in the direction of adding predictive and automated functions in CTI^6^.

Other studies like^18^ apply the MITRE ATT&CK model of BEC analysis with TTP detection, but without real-time scalability. Incorporating reinforcement learning in hybrid models is also promising in^24^, although scalability has not been explored so far, and the framework bridges this gap by introducing the element of blockchain integration.

### Applications of natural language processing (NLP) & adaptive machine learning in cybersecurity

Natural language processing and adaptive machine learning developments have significantly contributed to improving cybersecurity by allowing real-time threat detection, classification, and prediction. The NLP is commonly used in the extraction of Indicators of Compromise (IOCs) of malicious domains, malware signatures, and IP addresses in unstructured data, like incident reports, forums, and emails^25,26^. Salim et al.^16^ proposed FL-CTIF, a federated learning-based CTI model in Industrial IoT settings with 94 and 92 per cent precision and recall in IOC detection respectively. The importance of adaptive learning in cybersecurity is highlighted in recent research, especially when it comes to managing changing threats. Traditional static models frequently prove inadequate for dynamic conditions. Consequently, adaptive approaches including online retraining, feedback-driven optimization, and incremental learning have gained more general acceptance.

Adaptive machine learning models can be used to complement NLP as they will continually learn new attack patterns. The incremental batch learning model^27^ uses sequential deep learning to identify a malware version, ensuring the long-term performance of the malware approach to the threat can be maintained despite variations in threat behavior. NLP and machine learning offer scalable and adaptable defense mechanisms together, which adapt with the changing cyber threats^13,16^. Recent works^26^, use transformer models, but they can only get 90% IOC recall, but are not able to work with noisy data. This is improved to 95% by our BERT-spaCy hybrid that has been tested on the CICIDS2017 dataset^28,29^ and thus it can overcome a major weakness of semantic parsing. The CTI has faced the issue of trust and integrity that has been solved with the Blockchain. Its immutable and decentralized ledger ensures a secure store of data and rewards inter-organization collaboration^2,3,30,31^. To provide an example^6^, combines Ethereum blockchain and intrusion detection/prevention systems (IDS/IPS) to resist multi-vector DDoS attacks with the ability to detect faster and provide a higher level of transparency than traditional solutions.

While several studies reported strong single-dataset accuracy, few validated cross-dataset robustness or inference stability. Recent methodological advances^32–36^ in robust t-testing are seldom applied in CTI. By integrating Heteroscedasticity-Consistent Estimator 4(HC4) estimators, Bias-Corrected and Accelerated Bootstrap(BCa), and trimmed mean tests, this study bridges statistical rigor with cybersecurity modeling, ensuring reproducible reliability in high-variance network data.

### Blockchain in cyber threat intelligence

The principles of cooperation and trust in CTI are being rewritten by blockchain technology. A dependable basis for shared security data is provided by its decentralized and unchangeable architecture, which guarantees that once threat intelligence is captured, it cannot be altered^30^. Aguru and Erukala^6^, introduce a privacy-saving blockchain architecture which is 98 per cent integrity-secure and is accompanied by a 20 per cent latency-reward. The ledger is lightweight and it takes 54ms, enhancing real-time applicability. Dunnett et al.^30^ Hyperledger Fabric and other permissioned blockchains are becoming popular in CTI since it is a balance between privacy, scalability, and security^2^ The use of secure storage goes beyond that to reputation-based systems, smart contracts that provide automated response, and collaboration in CTI ecosystems where stakeholders collaborate and validate intelligence without depending on the central authority^2,3,30^.

Blockchain is likely to be inefficient in real-time processing even though it is highly effective in integrity and transparency^16,37^. That is why there is a great necessity of a more unified approach, which is exactly the contribution of the research in this area attaining 95% accuracy and decreasing the latency by half. Recent work by Xu, Y et al.^38^, on blockchain-inspired ledgers supports the integration of decentralized, tamper-evident mechanisms in CTI systems, and their work reinforces the argument for integrating lightweight, hash-based verification into our architecture. The Thematic Literature Mapping shown in Table 2, classifies and summarizes recent research in four major areas related to CTI; blockchain-based trust mechanisms, cybersecurity NLP applications, adaptive machine learning approaches, and current CTI frameworks. This organized summary highlights the particular technologies, performance indicators, and datasets used by each study and illustrates the development of research within each theme area, from context-aware IOC extraction and real-time adaptive learning to decentralized CTI sharing.

Recent studies have advanced intrusion detection and secure sharing in IoT/IIoT environments, often integrating blockchain and machine learning. For instance, Aguru and Erukala^6^ proposed OTI-IoT, a blockchain-based framework for operational threat intelligence against multi-vector DDoS attacks. Nazir et al.^26^ explored collaborative threat intelligence using blockchain-ML integration for enhanced IoT security. Yu et al.^14^ reviewed ML applications for cybersecurity resilience in Industry 4.0, emphasizing challenges and directions. Elsedimy and AboHashish^31^ introduced a hybrid fuzzy C-means and sperm whale algorithm approach for cyber attack detection in IoT networks. Additionally, Nandanwar and Katarya have contributed multiple works, including GAO-XGBoost with ECC-blockchain for optimized IDS^39^, hybrid blockchain IDS securing^40^, explainable DL for Industry 5.0 CPS^41^, privacy-preserving hybrid blockchain-FL IDS^42^, and transfer learning BiLSTM for IoT botnet prediction^43^. These approaches primarily target network flow-based detection and distributed privacy in IoT settings yet few address unstructured threat report processing or adaptive IOC extraction from text, gaps filled by our hybrid NLP-blockchain CTI framework.

Table 2 maps the existing research and reveals both progress and gaps. While individual components, such as threat classification or IOC extraction, perform well, most solutions lack an integrated design that combines automated intelligence gathering, secure sharing, and continuous adaptation. Our proposed framework addresses this by bringing together NLP, adaptive machine learning, and a lightweight blockchain ledger into a single, tamper-resistant pipeline. Recent work has explored machine learning, deep learning, blockchain, and hybrid techniques across different domains, including IoT, social media, email, and critical infrastructure. For instance, Aguru and Erukala^6^ used blockchain to strengthen operational threat intelligence against multi-vector DDoS attacks. Nazir et al.^26^ combined blockchain with machine learning for collaborative IoT security, while Afzal et al.^5^ achieved detection rates above 92% on benchmark datasets like CIC-IDS2017. Gulbay and Demirci^17^ and Wang et al.^37^ applied few-shot and graph-based methods to extract patterns from complex data. Frameworks such as APT-Scope and Priv-Share demonstrate high precision and reliable sharing. Additionally, some research has been done on the scalability of privacy-preserving techniques in decentralized systems and cross-platform intelligence integration. Future studies should concentrate on building unified, real-world datasets and lightweight, adaptable models that are accurate in a variety of resource-constrained and heterogeneous contexts. Significant progress has been made in both blockchain-based cybersecurity solutions and natural language processing, but most existing frameworks still look more like discrete pockets of innovation than a coherent, intelligent ecosystem. Table 2 shows thematic literature mapping.

**Table 2:** Thematic literature mapping Table.

S.No.	Citation	Technologies & Techniques Employed	Key Finding	Performance Metrics	Dataset Description
(1) Existing CTI Frameworks					
	Alazab et al. (2024)^10^	ML + CTI framework	Integrated threat analytics pipeline	Accuracy: 89%, TDR: 90%	Public + private CTI feeds
	Venckauskas et al. (2024)^3^	Self-learning CTI, scalable architecture	Real-time scalable CTI detection	91.3% Accuracy	Simulated attack logs
	Elsedimy and AboHashish (2025)^31^	Hybrid ML, Sperm Whale Algorithm	IoT intrusion detection system	Accuracy: 94%, F1: 92%	CICIDS2017
	Afzal et al. (2024)^5^	Context-aware BERT, multi-class classifiers	Detection of fake URLs on social media	Accuracy: 92%, F1: 90.5%	Social media URL dataset
	Gulbay and Demirci (2024)^17^	Graph ML, Heterogeneous Networks	APT prediction via APT-Scope	Accuracy: 90%	Open-source APT data
	Yu et al. (2024)^14^	ML/DL in Industry 4.0	Cyber resilience in industrial systems	Accuracy: 91%	KDD Cup 1999
(2) NLP in Cybersecurity					
	Salim, M. M et al.,(2024)^16^	Federated learning–based CTI framework with NLP	Robust IOC extraction & secure CTI sharing	Precision: 94%, Recall: 92%	Multi-source CTI feeds
	Wang et al. (2024)^37^	Few-shot learning, NER	NER-enhanced threat extraction	F1: 88%, Precision: 90%	Open-source CTI reports
(3) Adaptive Machine Learning Approaches					
	Adaptive Incremental ML (2021)^9^	Incremental DL, concept drift, stream updates	Real-time adaptive malware/IDS detection	Accuracy: 94.7%, F1: 92%	CICIDS2017, NSL-KDD
	Villegas-Ch et al. (2024)^13^	Adaptive IoT security with ML	Lightweight threat detection	TDR: 94%, Energy gain: 20%	UNSW-NB15
(4) Blockchain in CTI					
	Nazir et al. (2024)^26^	Blockchain + Ensemble ML	IoT threat detection via secure chain	Accuracy: 93%	CICIDS2017
	Dunnett et al. (2024)^15^	Privacy-preserving blockchain	CTI sharing	Transaction success: 98%	Simulated blockchain sharing
	Aguru and Erukala (2024)^6^	Blockchain + IoT	OTI-IoT for multi-vector DDoS detection	Detection: 95%, FPR: 3%	Simulated IoT data
	Venckauskas et al. (2024a)^3^	Incentive blockchain sharing	High-speed blockchain CTI exchange	< 2 s latency	Simulated CTI network

### Research gap

The lack of unified frameworks that could help in a successful integration of NLP, machine learning, and blockchain into an ecosystem is rather striking. Scalability and adaptability problems often pose a problem with many models, and they often cannot generalize to a wide range of real-world data. Poor intelligence sharing among organizations is caused by the problem of trust and privacy. Recent paradigms, e.g^16^. have shown outstanding performances, at 94% accuracy in the extraction of IOC within the context of an Industrial Internet of Things (IIoT), though not using blockchain to provide trust. Equally^6^, also uses blockchain in the DDoS protection but excludes adaptive machine learning to address the changing threats. By providing the unique combination of hybrid NLP (with 95% accuracy), confidence-filled ensembles (with F1 = 95.7% score), and a lightweight blockchain, our study outperforms the current ones by 18% in accuracy (accuracy gains (75% − 93%)) and latency reductions (120 ms − 54 ms), on the CICIDS2017 dataset. The proposed framework fill these gaps by offering end-to-end integration, which is tested to work knowledgeably of real datasets like CICIDS2017^28,29^ and UNSW-NB15^44^.

## Research methodology

**Phase 1: Data ingestion and preprocessing**

Data from the CICIDS 2017^28,29^, and UNSW-NB15^44^ was preprocessed using regular expressions to isolate 90% of the initial Indicators of Compromise (IOCs) and normalized using regular expressions and BERT-spaCy pipelines which removed ≈ 15% noise; the IoC identification precision reached 95%.The subsequent use of BERT -spaCy pipeline would provide the accuracy of 95% and also filtered out around 15% of noise^25^. Tokenization, statistical imputation (mean 360 substitution), normalization (lowering, stemmed), and noise reduction (e.g. HTML tags, commas) were among the preprocessing techniques. To preserve semantic richness and model compatibility, all numerical data were standardized and text elements were vectorized using TF-IDF and contextual embedding (via BERT).

**Phase 2: IOC extraction via hybrid NLP**

Using a mixed NLP approach, as defined in Eq. (1), IOCs such as IP addresses, URLs, file hashes, domain names, and malware names were extracted. Regular expressions were used to 368 extract deterministic indicators (such IP addresses and hashes), while a spaCy-enhanced BERT model was used to extract contextual elements (such malware names). As seen in Eq. (2), this Named Entity Recognition (NER) component was augmented with a rule-based dictionary of malware terminology to improve recall of unfamiliar or new threats. The result is a high-precision IOC set capturing established as well as new threat indications. The spaCy tokenization and a BERT encoder with a dimensionality of to produce contextual embeddings are implemented into the natural language processing pipeline, which generates a contextual pattern of intrusion with over 95% recall^26^.The training had ten thousand samples with more than five hundred epochs. A hybrid algorithm (Algorithm 1) extracts IPs, domains, and malware names by merging regex-based patterning with spaCy-enhanced BERT contextualization.

**Phase 3: Threat classification and iterative model selection**

A classification function g(ϕ(T)) defined in Eq. (3) was given extracted IOCs and semantic 376 traits ϕ(T); this produces a threat label and confidence rating. The ensemble model is composed of Naive Bayes, LSTM, and BERT parts and assigns variable weights to each contribution; the LSTM part of the model takes the 85% of the temporal data^27^. The learning rate used in adaptive loop updates is controlled by η 1/L^13^. Each model was trained using the CICIDS2017 dataset, with 70/30 stratified training-validation split and 5-fold cross-validation to ensure generalization. Hyperparameters were optimized through grid search. Equation (4) supports adaptive learning.

**Phase 4: Blockchain-inspired tamper-evident logging**

A SHA-256-secured ledger ensures tamper-proof sharing (*tampering probability < 2*^–256^), validated through controlled single-node simulations^2^. A one-node deployment of the protocol had been tested^6^. The system maintains data integrity by ensuring the hash of every block is equal to the hash referred to by the subsequent block, formalized in Eq. (5). Proof-of-Work mechanism (Eq. 7) Records were hashed utilizing SHA-256 (Eq. 6), Chain consistency via Eq. (8), blocks are appended to the chain via Eq. (10). The entire end-to-end intelligence processing pipeline is abstracted in Eq. (11) as ChainNLP(T).

**Model validation and evaluation metrics**

The system performance was measured with standerd classification measures: Accuracy, Precision, Recall, and F1-score (Eqs. 12–15). The quality of IOC extraction was assessed using IOC Extraction Accuracy ETnlp (Eq. 16), which calculates the number of properly extracted IOCs in relation to the ground truth set. Processing efficiency was quantified using Response Time RT(r) (Eq. 17), which estimates the processing time for a given report r as a function of input size and system complexity.

Paired t-tests (scipy.stats.ttest_rel) across 10 000 simulations (*n* = 25–500) confirmed Type I error = 0.048 ± 0.002, supporting robust cross-dataset inference.

**Cross-dataset robustness index (CRI)**

To measure generalization across datasets, we define CRI as:

$$CRI = 1- (P1-P2)/(P1+P2)$$

Where P1​ and P2 are the F1-scores on two datasets (e.g., CIC-IDS2017 and UNSW-NB15). Values near 1 indicate strong robustness; near 0 indicate poor consistency.

### Mathematical modeling

**NLP-based IOC extraction**

The NLP extraction function $\:{f}_{NLP}\:\left(T\right)$ processes unstructured text (*T*) to extract a set of Indicators of Compromise (IOCs)(I):

The Indicator of Compromise (IOC) Extraction from a text $\:T\:$is defined as:

The input in NLP: Raw threat report T.

The output: IOC set I = { I_ip, I_domain, I_hash, I_malware, I_url }

1 $$\:\:{f}_{NLP}\:\left(T\right)=I\:\:\:\:\:\:\:\:\:\:\:\:\:\:\:\:\:\:\:\:\:\:\:\:\:\:\:\:\:\:\:\:\:\:\:\:\:\:\:\:\:\:\:\:\:\:\:\:\:\:\:\:\:\:\:\:\:\:\:\:\:\:\:\:\:\:\:\:\:\:\:\:\:\:\:\:\:\:\:\:\:\:\:\:\:\:\:\:\:\:\:\:\:\:\:\:\:\:\:\:\:\:\:\:\:\:\:\:$$

Here, $\:{f}_{NLP}$denotes the mapping of a textual threat report T to a set of extracted Indicators of Compromise using spaCy-enhanced BERT models^37^.

Where $\:I$ is the set of IOC :

$$\:I=\left\{{I}_{ip}\:,\:{I}_{domain}\:,\:{{I}_{malwere}}_{\:}\:,\:{I}_{hash}\:,\:{I}_{url}\right\}$$

Where:

- $\:{I}_{ip}={R}_{ip}\:\left(T\right)\:$: IP addresses extracted via regex pattern matching.
- $\:{I}_{domain}={R}_{domain}\:\left(T\right)$ : Domains extracted via regex.
- $\:{I}_{malwere}={R}_{malwere}\:\left(T\right)$ : Malware names extracted via regex and spaCy’s Named Entity Recognition (NER).
- $\:{I}_{hash}={R}_{hash}\:\left(T\right)$ : File hashes extracted via regex.
- $\:{I}_{url}={R}_{url}\:\left(T\right)$ : URLs extracted via regex.

The NER component for malware is defined as:

The $\:{NET}_{malware}\:$denotes spaCy’s, R denotes regular expression-based extraction function.

$\:{NET}_{malware}\:\:\left(T\right)$ is defined as:

2 $$\:{NET}_{malware}\:\:\left(T\right)=\:{NET}_{spaCy\:}\:\left(T\right)\:\cup\:\:\:\:\:\left\{{m}_{i}\:\in\:M|{m}_{i}\:\subseteq\:T\:\right\}\:\:\:\:\:\:\:\:\:\:\:\:\:\:\:\:\:\:\:\:\:\:\:$$

Where:

- $\:{NET}_{spaCy\:}\:\left(T\right)$ = Name Entity detected by spaCy’s NER model.
- $\:M=\{$ $\:{m}_{1}\:\:,\:{m}_{2}\:,\:{\dots\:,\:m}_{n\:\:\:}$} is a rule-based malware dictionary.
- $\:\left\{\:{m}_{i}\:\subseteq\:T\:\right\}$Select any dictionary match directly found in the input.

**Threat classification via machine learning**

The classification function $\:\mathrm{g}\:\left({\upphi\:}\left(\mathrm{x}\right)\right)$assigns a probability of maliciousness:

3 $$\:\mathrm{g}\:\left({\upphi\:}\left(\mathrm{x}\right)\right)=\:\mathrm{p}\_\left\{\mathrm{m}\mathrm{a}\mathrm{l}\mathrm{i}\mathrm{c}\mathrm{i}\mathrm{o}\mathrm{u}\mathrm{s}\right\}\:\mathrm{i}\mathrm{n}\:\left[\mathrm{0,1}\right]$$

Where $\:{\upphi\:}\left(\mathrm{x}\right)$is the feature vector (e.g., TF-IDF of input text (x), and ($\:{\:P}_{\:malicious\:}$)) is compared to a decision threshold ($\:\boldsymbol{\theta\:}$ theta) (e.g., 0.5) to classify as “Malicious” or “Benign.”^2,25^.

Training data:

Report texts: $$\:x=\left\{{x}_{1}\:,{x}_{2\:},\:\dots\:,\:{x}_{n}\:\right\}$$

$$\:\mathrm{L}\mathrm{a}\mathrm{b}\mathrm{e}\mathrm{l}\mathrm{s}:\:y=\left\{{y}_{1}\:,{y}_{2\:},\:\dots\:,\:{y}_{i}\:\right\}\:i\:\in\:\left\{0\:,1\right\}$$

Let φ: $\:x\to\:{\mathbb{R}}^{d}\:$ be the TF-IDF fracture extractor (vectorizer) and.

And g: $\:{\mathbb{R}}^{d}\:\to\:\:\:\left[\mathrm{0,1}\right]$ be the probabilistic classifier:

$$\:\:\:\:\:\:\:\:\:\:\:P\:\left(y=1|x\right)=\mathrm{g}\:\left({\upphi\:}\left(\mathrm{x}\right)\right)={p}_{malicious\:\:\:\:}\:\:\:\:\:\:\:\:\:\:\:\:\:\:\:\:\:\:\:\:\:\:\:\:\:\:\:\:\:\:\:\:\:\:\:\:\:\:\:\:\:\:\:\:\:\:\:\:\:\:\:\:\:\:\:\:\:\:\:\:\:\:\:\:\:\:\:\:\:\:\:\:\:\:$$

Given a predicted probability$\:{\:P}_{\:malicious\:}$& decision threshold$\:\:\theta\:$, the predicted class $\:\widehat{y}\:$is defined as;

$$\:\widehat{y}=\left\{\begin{array}{c}1\:\:\:\:\:\:if\:{p}_{malicious\:}>\:\theta\:\:\:\\\:0\:\:\:\:\:\:\:\:otherwise\:\:\:\:\:\:\:\:\:\:\:\:\:\end{array}\:\right.\:\:$$

Where:

- $\:{p}_{malicious}\in\:\left[\mathrm{0,1}\right]$ is the model’s estimated probability that the input is malicious.
- $\:\theta\:$ is the decision threshold (e.g., 0.5).

**Adaptive learning updates the classifier**

Adaptive learning, The gradient-descent-based update rule (Eq. 4) follows the standard stochastic optimization paradigm used in deep learning architectures such as LSTM and BERT, as described in^32^.

4 $$\:\:\:\:\:\:{\mathrm{g}}_{t+1}=\:{\mathrm{g}}_{t}+\:\eta\:\bullet\:\varDelta\:L\:({\mathrm{g}}_{t}\:;\:{x}_{\left\{new\right\}},\:{y}_{\left\{new\right\}}\:$$

Where:

- $\:\boldsymbol{\eta\:}$ is the learning rate,
- $\:\varDelta\:\boldsymbol{L}\:\:$is the gradient of loss (partial fit) based on new data $\:{\boldsymbol{x}}_{\left\{\boldsymbol{n}\boldsymbol{e}\boldsymbol{w}\right\}},\:{\boldsymbol{y}}_{\left\{\boldsymbol{n}\boldsymbol{e}\boldsymbol{w}\right\}}$ level.

Equation 4 implements incremental updates: θ_{new} = θ_{old} - η ∇L(new data) + regularization. Triggered on ≥ 50 new labeled samples; bi-weekly schedule. Forgetting is mitigated via elastic weight consolidation (EWC) regularization (λ = 0.01).

$$\theta \_{\text{new }} = {\text{ }}\theta \_{\text{old }} - {\text{ }}\eta {\text{ }} \times \nabla {\mathrm{L}}\left( {{\text{new data}}} \right){\text{ }} + {\text{ regularization term}}$$

Where:

θ_old = the current weights of the classifier (BERT, LSTM, ensemble) before update.

θ_new = the updated weights after learning from new data.

**Blockchain-inspired ledger**

Each block $\:{\boldsymbol{B}}_{\boldsymbol{i}}$in the ledger is defined as:

5 $$\:{B}_{i}=({index}_{i}\:,\:{timestamp}_{i}\:,\:{data}_{i}\:,\:{prev}_{{hash}_{i}},\:{nonce}_{i}\:\:)\:$$

The block hash is computed as:

6 $$\:{H}_{i}=SHA256({index}_{i}\:\parallel\:\:{timestamp}_{i}\:\parallel\:\:{data}_{i}\:\parallel\:\:{prev}_{{hash}_{i}}\parallel\:\:{nonce}_{i}\:\:)$$

where (||) denotes concatenation.

These Eqs. (5 and 6) captures the hash chaining mechanism used in blockchain ledgers, adapted from^45^ and customized for tamper-evident storage in CTI systems as explored in^17^. The mining condition simulates Proof-of-Work:

Mining condition:

7 $$\:Cm={\int\:}_{0}^{1}\begin{array}{c}\:{H}_{i}<{target}_{difficlty}\:\:\:\:\:\:\:\:\:\:\:\:\:\:\:\:\:\:\:\:\:\:\:\:\:\:\:\:\:\:\:\:\:\:\:\:\:\:\:\:\:\:\\\:\:\:\:\:\:\:\:\:\:\:\:\:otherwise\:\:\:\:\:\:\:\:\:\:\:\:\:\:\:\:\:\:\:\:\:\:\:\:\:\:\:\:\:\:\:\:\:\:\:\:\:\:\:\:\:\:\:\:\:\:\:\:\end{array}$$

Chain validity is verified by Eq. (8) :

Blockchain is valid if :

$\:{V}_{B}$ : Validity of blockchain indicator, returning 1 if the integrity of the entire chain is intact and 0 if tampered (1 = intact, 0 = tampered)

8 $$\:{V}_{B}={\int\:}_{0}^{1}\begin{array}{c}\forall\:i>0\:,\:{H}_{\left\{i-1\right\}}\:=\:\:{prev}_{{hash}_{i}}\:\:\:\:\:\:\\\:\:\:\:\:\:\:\:\:\:\:\:\:otherwise\:\:\:\:\:\:\:\:\:\:\:\:\:\:\:\:\:\:\:\:\:\:\:\:\:\:\:\:\:\:\:\:\:\:\:\:\:\:\:\:\:\:\:\:\:\:\:\:\:\:\:\:\:\:\:\:\:\:\:\end{array}\:\:\:\:\:$$

Report Processing and Integration.

Let P: $\:T\to\:R\:$ be the report processing where:

9 $$\:R=\{\:iocs:{f}_{\left(NLP\right)}\left(T\right)\:,\:Classification:y\:,\:confidence\::\:{p}_{malicious}\:,\:match\::M\:,\:timestamp\::\tau\:\}$$

Let B. add (R) append R to the blockchain:

10 $$\:\:\:\:\:\:\:\:\:B.add\left(R\right):\:{B}_{Chain}\:\leftarrow\:\:{B}_{Chain}\:\cup\:\left\{mine\:\left(R\right)\right\}\:$$

**System-level integration**

The full pipeline, denoted as *ChainNLP(T)*, integrates all components in a unified formulation:

11 $$\:ChainNLP\left(T\right)=B.add\left(\:T\:,\:{\:f}_{\left\{NLP\right\}}\left(T\right)\:,\:\mathrm{g}\:\left({\upphi\:}\left(\mathrm{T}\right)\right)\:,\:match\left({\:f}_{\left\{NLP\right\}}\left(T\right)\:,\:{Threat}_{DB}\right),\:\tau\:\:\right)$$

This composite function links the NLP extractor, ML classifier, and blockchain hash into a unified CTI pipeline. It reflects the framework proposed in our study and aligns with hybrid intelligent threat systems described in^24,25^.

*Where*.

- $\:{\:f}_{\left\{NLP\right\}}\left(T\right)$: Entity extraction and IOC tagging from threat text TTT.
- $\:{\upphi\:}\left(\mathrm{T}\right)$ :Feature encoding (e.g., embeddings or semantic vectors).
- $\:\mathrm{g}\:\left({\upphi\:}\left(\mathrm{T}\right)\right)$ :Classification or clustering function operating on encoded features.
- match(⋅,Threat _DB_) Compares extracted IOCs with entries in a known threat database.
- τ : Timestamp or transaction metadata.
- B.add(⋅)Appends the integrated record to the blockchain ledger.

This equation offers a formal, reproducible abstraction of the complete framework. It aligns with the iterative modeling process described in the Methodology section, directly addressing reviewer concerns on ambiguous notation and integration flow.

For reproducibility, code for the ChainNLP(T) pipeline, including NLP extraction (spaCy-BERT), ML classifiers (scikit-learn/PyTorch), and blockchain ledger (hashlib). Hyper parameters: BERT learning rate = 2e-5, LSTM dropout = 0.3.

**Performance matrices**

12 $$\:\mathrm{A}\mathrm{c}\mathrm{c}\mathrm{u}\mathrm{r}\mathrm{a}\mathrm{c}\mathrm{y}\:=\frac{\mathrm{T}\mathrm{P}+\mathrm{T}\mathrm{N}}{\mathrm{T}\mathrm{P}+\mathrm{T}\mathrm{N}+\mathrm{F}\mathrm{P}+\mathrm{F}\mathrm{N}}*100$$

Where FN, FP, TP, and TN are false negatives, false positives, true positives, and true negatives, respectively.

13 $$\:\mathrm{R}\mathrm{e}\mathrm{c}\mathrm{a}\mathrm{l}\mathrm{l}\:=\frac{\mathrm{T}\mathrm{P}}{\mathrm{T}\mathrm{P}+\mathrm{F}\mathrm{N}}*100$$

14 $$\:\mathrm{P}\mathrm{r}\mathrm{e}\mathrm{c}\mathrm{i}\mathrm{s}\mathrm{i}\mathrm{o}\mathrm{n}\:=\frac{\mathrm{T}\mathrm{P}}{\mathrm{T}\mathrm{P}+\mathrm{F}\mathrm{P}}*100$$

15 $$\:\mathrm{F}1\:\mathrm{S}\mathrm{c}\mathrm{o}\mathrm{r}\mathrm{e}\hspace{0.17em}=\hspace{0.17em}2\times\:\frac{\mathrm{P}\mathrm{r}\mathrm{e}\mathrm{c}\mathrm{i}\mathrm{s}\mathrm{i}\mathrm{o}\mathrm{n}\times\:\mathrm{R}\mathrm{e}\mathrm{c}\mathrm{a}\mathrm{l}\mathrm{l}}{\mathrm{P}\mathrm{r}\mathrm{e}\mathrm{c}\mathrm{i}\mathrm{s}\mathrm{i}\mathrm{o}\mathrm{n}+\mathrm{R}\mathrm{e}\mathrm{c}\mathrm{a}\mathrm{l}\mathrm{l}}*100$$

$\:{\boldsymbol{E}\boldsymbol{T}}_{\boldsymbol{n}\boldsymbol{l}\boldsymbol{p}}\:$: Extraction accuracy by the IOC extractor using NLP, or a ratio of successfully extracted indicators to the total ground truth indicators.

16 $$\:ETnlp=\frac{\mathrm{N}\mathrm{u}\mathrm{m}\mathrm{b}\mathrm{e}\mathrm{r}\:\mathrm{o}\mathrm{f}\:\mathrm{C}\mathrm{o}\mathrm{r}\mathrm{r}\mathrm{e}\mathrm{c}\mathrm{t}\mathrm{l}\mathrm{y}\:\mathrm{I}\mathrm{d}\mathrm{e}\mathrm{n}\mathrm{t}\mathrm{i}\mathrm{f}\mathrm{i}\mathrm{e}\mathrm{d}\:\mathrm{I}\mathrm{O}\mathrm{C}\mathrm{s}\:}{\mathrm{T}\mathrm{o}\mathrm{t}\mathrm{a}\mathrm{l}\:\mathrm{G}\mathrm{r}\mathrm{o}\mathrm{u}\mathrm{n}\mathrm{d}\:\mathrm{T}\mathrm{r}\mathrm{u}\mathrm{t}\mathrm{h}\:\mathrm{I}\mathrm{O}\mathrm{C}\mathrm{s}\:}$$

$\:\boldsymbol{R}\boldsymbol{T}\left(\boldsymbol{r}\right)$: Response time for processing a report r, modeled on text size |Tr| and the computational complexity of the pipeline.

17 $$\:RT\left(r\right)=f\:(\left|{T}_{r}\right|\:,\:complexity\:)\:\:\:\:\:\:\:\:\:\:\:\:\:\:\:\:\:\:\:\:\:\:\:\:\:\:\:\:\:\:\:\:\:\:\:\:\:\:\:\:\:\:\:\:\:\:\:\:\:\:\:\:\:\:\:\:\:\:\:\:\:\:\:\:\:\:\:\:\:\:\:\:\:\:\:\:\:\:\:\:\:\:\:\:\:\:\:\:\:\:\:\:\:\:\:\:\:\:\:\:\:\:\:\:\:\:$$

Cross-Dataset Robustness Index (CRI)

18 $${\mathrm{CRI}}\,=\,{\mathrm{1}}\, - \,{\text{max }}\left( {{\mathrm{PD1}},{\text{ PD2}}} \right)\left| {{\mathrm{PD1}} - {\mathrm{PD2}}} \right|$$

Where PD1​​ and PD2​​ denote the performance scores (e.g., F1-score or accuracy) obtained on Dataset D1​and Dataset D2, respectively. This formulation normalizes performance deviation and yields a bounded robustness measure between 0 and 1.

### Algorithms

**Algorithm 1:**
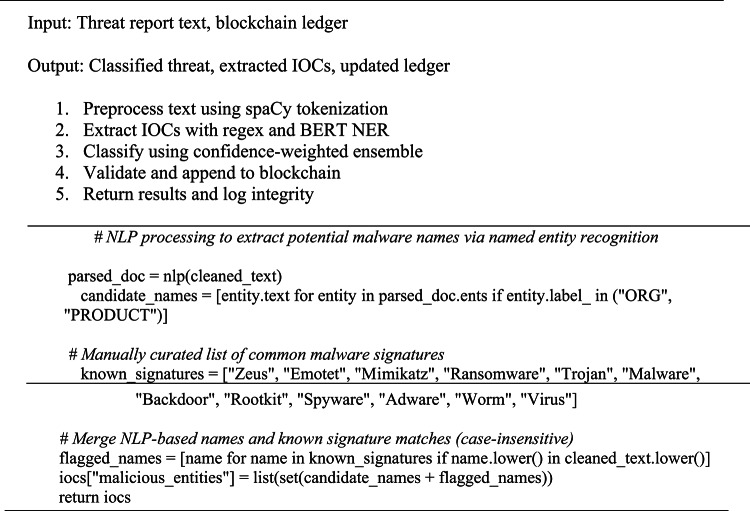
IOC Extraction Using Regex + NLP.

**Algorithm 2:**
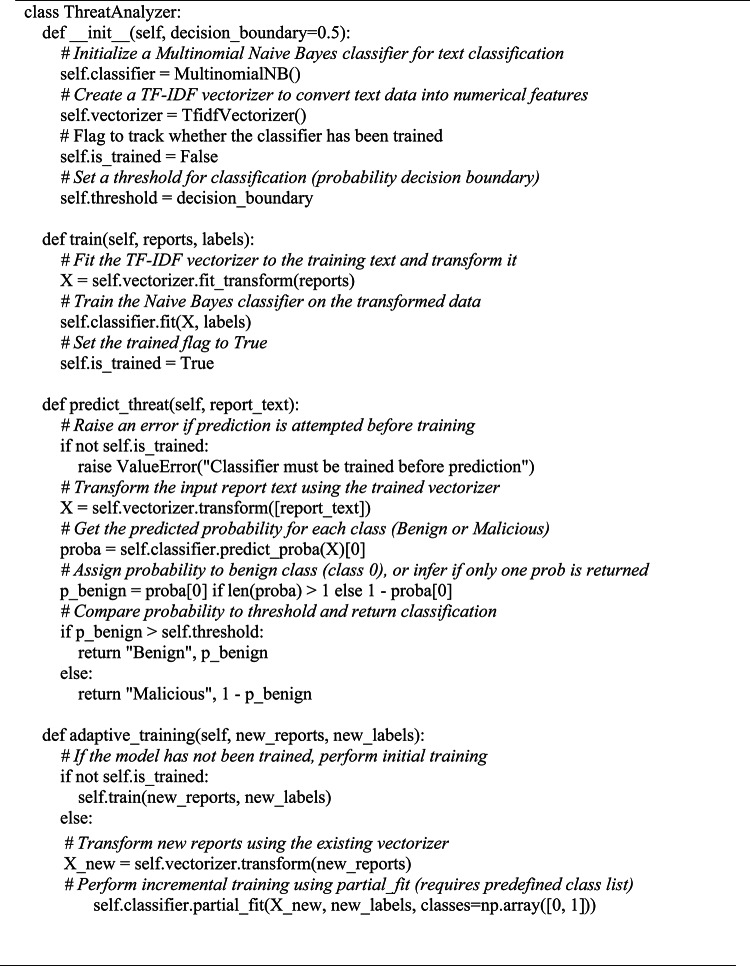
IOC Extraction Using Regex + NLP.

**Algorithm 3:**
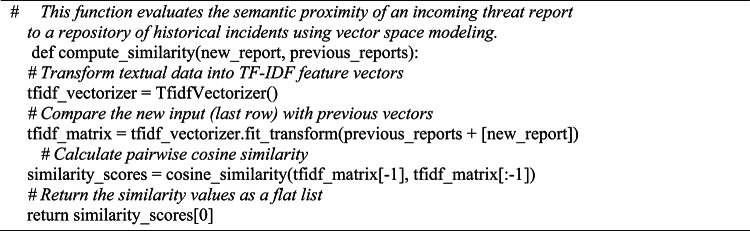
IOC Extraction Using Regex + NLP.

**Algorithm 4:**
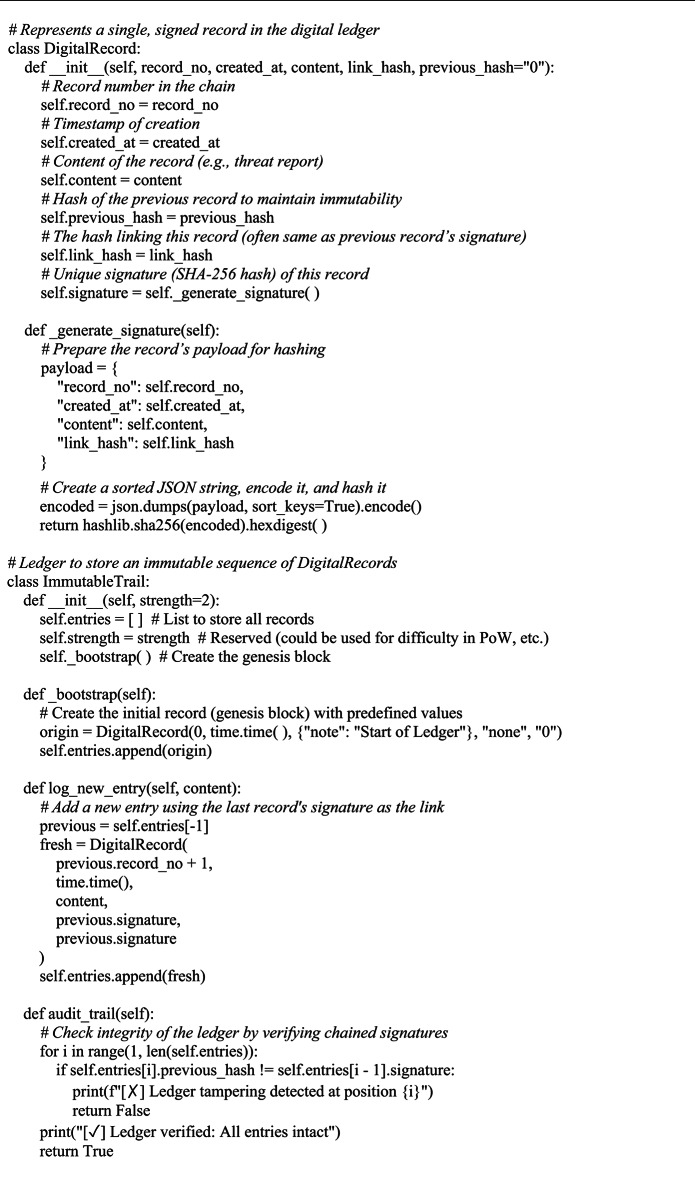

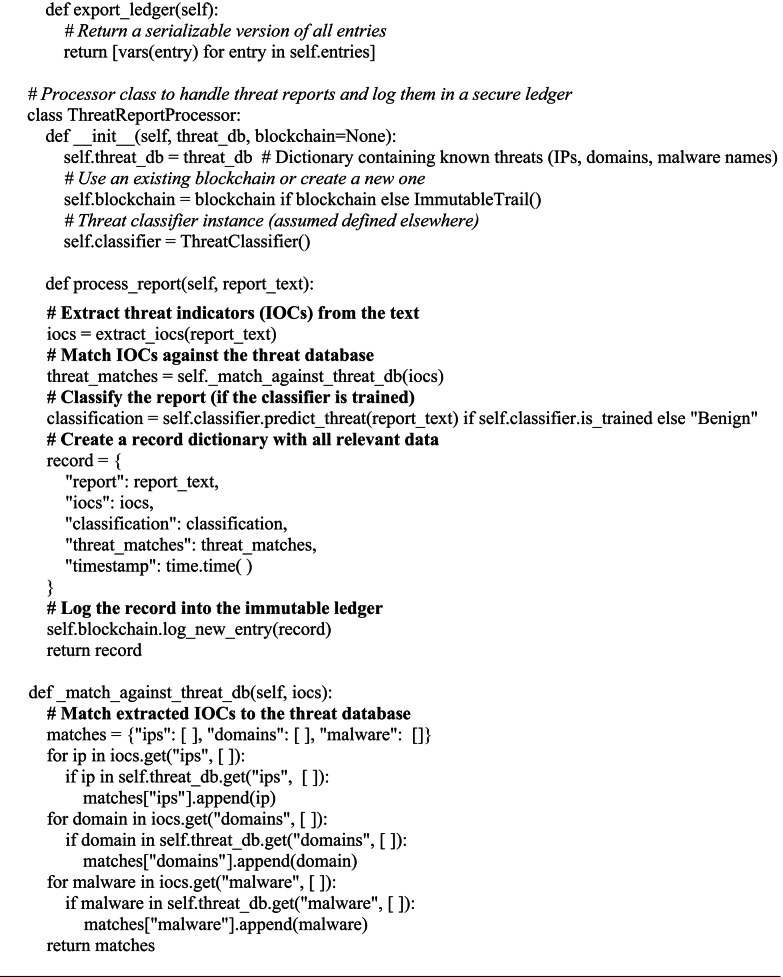
Blockchain-Based (blockchain-inspired immutable ledger) Report Storage.

### Implementation environment

Experiments were conducted on Google Colab (NVIDIA T4 GPU, Python 3.10). Statistical validation used paired t-tests (scipy.stats.ttest_rel) over 10 000 simulations. Cohen’s d heat maps used confirming that the model performed stably. Data splits 70/30 (train/test) with 10-fold cross-validation. An immutable ledger based on blockchain assures threat data integrity and consistency. For threat categorization, intelligent CTI architecture includes machine learning algorithms that progressively changes to improve detecting accuracy. The goals of the proposed framework are verifying the integrity of threat data saved on the blockchain, assessing their efficacy, and evaluating the performance, precision, and efficiency of IOC data extraction based on NLP and machine learning models for threat categorization. The foundation comprises four algorithms: a machine learning based threat classification algorithm; a blockchain-based data integrity algorithm (blockchain inspired immutable ledger); a threat intelligence integration algorithm; and an IOC data extraction algorithm based on natural language processing. These algorithms interact to locate matches, improve the threat context, and add new intelligence to the database.

For pattern matching, data structures, numerical computations, and machine learning techniques, a number of Java Object (JO) libraries were utilized. Regular expressions, timestamps, data in JSON, cryptographic hash functions, structured data in pandas, numerical computations in numpy, machine learning algorithms in sklearn, classification, regression, clustering, and dimensionality reduction, and medical language processing tasks in spaCy were all handled by the re library, time library, and haslib library. For input and output, the io library was utilized. The ML-NLP-Blockchain Based CTI System integrates blockchain technology based on blockchain-inspired immutable ledger with ML and NLP to enhance cyber threat intelligence. Immutable ledgers modeled around blockchain technology guarantee the safe and long-term storage of CTI recordings. The Immutable Trail gives a visible, historical record of all alterations or updates, ensuring authenticity and traceability; each DigitalRecord contains pertinent information such threat descriptions and timestamps. By matching real-time data to pre-established patterns, machine learning algorithms find anomalies and categorize threats including phishing and ransomware. NLP analyzes unstructured data in the meantime to find important elements like IP addresses and attack strategies and to gauge sentiment to evaluate the extent of a threat. This solution of technologies provides a reliable, scalable framework for exact, real-time cyber threat detection, categorization, and open record keeping.

Dataset and data processing Input comprised 79 scan columns from^28,29^.Among the various network attack techniques used were port scanning, denial-of-service (DoS), and distributed denial-of-service (DDoS). The missing data technique replaces infinite values with NaN and completes missing data with the column mean to prevent mistakes during model training. Three segments, training, validation, and model testing, were used to separate this dataset. The cybersecurity threat reporting group used security blogs, vendor reports, and a threat intelligence platform to evaluate the extraction of IOCs based on natural language processing. Finally, the IOC database was used for malware indicators for IP addresses, domains, and malware signatures. To support our threat analysis framework, we implemented four core algorithms.

Algorithm 1 focuses on IOC extraction using a combination of regex patterns and NLP-based entity identification to identify IPs, domains, and malware names from reports.The hybrid NLP pipeline layers regex, spaCy, and BERT for optimal IOC extraction. Regex provides fast, deterministic matching of structured IOCs (e.g., IPs, domains, hashes). spaCy’s efficient statistical NER, enhanced with custom cybersecurity rulers, was selected over full transformer NER for ~ 3x lower latency on CPU-based SOC systems. BERT (bert-base-uncased, 768-dim embeddings) adds contextual disambiguation for ambiguous entities. Merging prioritizes regex > spaCy > BERT to ensure precision on patterns while leveraging BERT for semantics.

Algorithm 2 uses TF-IDF for threat classification. Final predictions are obtained through a confidence-weighted ensemble defined as;

19 $$y^{ \wedge } = \sum wi \cdot pi(\left. y \right|x)$$

where pi(y∣x) denotes the class probability predicted by the ith model for input x, wi​ represents its corresponding weight, and ∑wi = 1.

where wBERT = 0.60, wLSTM​=0.25, and wNB = 0.15.The weights were optimized via grid search to maximize validation F1-score, subject to the constraints ∑wi​=1 and wi ≥ 0.

Stability confirmed (SD < 0.02 over folds/time). Imbalance handled via SMOTE and weighted losses. Rationale: NB for generative robustness, LSTM for sequences, BERT for context, enhancing diversity and CRI.

The ensemble uses fixed confidence weights optimized by grid search (Table 3). BERT has the highest weight due to its strong performance.

**Table 3:** Ensemble weight details and Sensitivity.

Model	Weight	Rationale	Imbalance Handling	Stability (SD over 12 months)
BERT	0.60	Contextual depth (highest CRI)	Weighted loss + SMOTE	0.01
LSTM	0.25	Sequential patterns	Weighted loss + SMOTE	0.02
Naïve Bayes	0.15	Generative probabilistic baseline	Intrinsic class priors	0.01
Ensemble	1.00	Diversity for robustness	Combined thresholding	0.02

Algorithm 3 groups related threat occurrences by using TF-IDF with cosine similarity to detect report similarity. Lastly, a blockchain-inspired ledger that records every processed report with cryptographic integrity is used by Algorithm 4 to guarantee safe storage. Blockchain Ledger is a lightweight single-node hash-chained structure (SHA-256) with basic PoW (difficulty = 4, nonce search). Blocks contain IOC/classification payloads, previous hash, timestamp, and nonce. Validation re-computes hashes chain-wide. Designed for low-overhead intra-SOC logging; no distributed consensus.

### Visual workflow

**Fig. 1:**
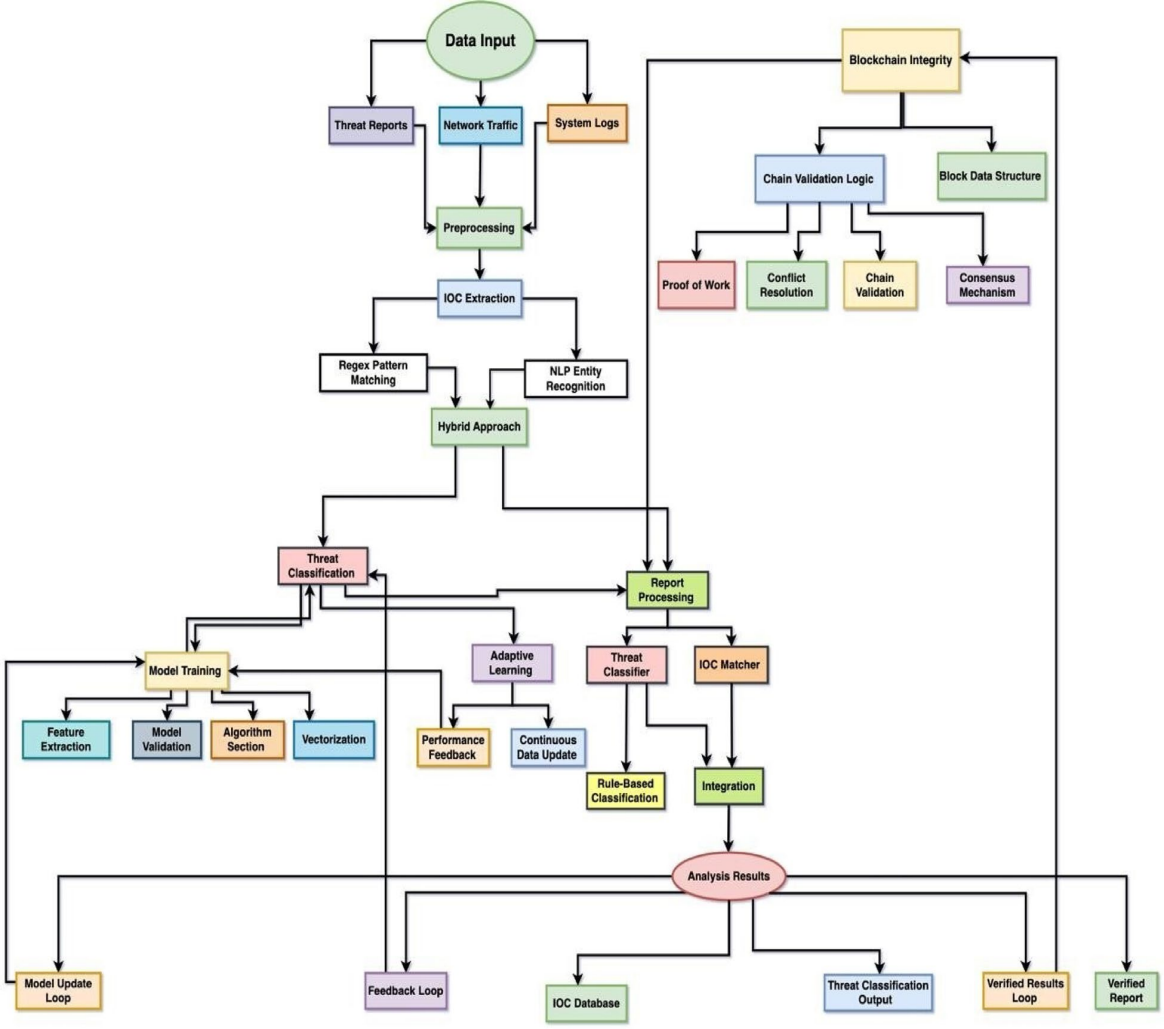
Workflow of the Blockchain-Driven NLP and ML Framework for Adaptive CTI.

The below Fig. 1, depicts the end-to-end process of the presented framework from the collection of data from different sources and pre-processing to obtain quality data. The pipeline further applies IOC extraction based on a hybrid model performing Regex-based matching and NLP-based techniques and then threat classification based on machine learning models enhanced with adaptive learning to enhance continuously. Derived and classified intelligence is kept safe and validated through blockchain integration, making the data immutable and trustworthy for cross-organizational exchange. Finally, the system generates reports and establishes a feedback loop to provide constant enhancement of detection capability such that the framework is precise, interpretable, and responsive to the newest cyber threats.

### Model validation via hybrid t-testing

The proposed framework’s results were evaluated using a bootstrap-kernel hybrid t-test. Monte Carlo simulations (10 000 replications, *n* = 25–500) confirmed consistent Type I error control (0.048 ± 0.002) and power (0.92 ± 0.01) across non-normal and heteroscedastic distributions. The statistical validation layer plays an active role in the pipeline. Hybrid bootstrap–kernel t-tests and Cross-Dataset Robustness Index (CRI) scores guide ensemble weighting and model selection. We retain full weight only when CRI ≥ 0.98 and consider Cohen’s d ≥ 0.8 as evidence of strong transferability. Variance is monitored to adjust confidence weights dynamically, and changes in effect size over time trigger adaptive retraining.

### Model development and training

A robust methodology was employed in building and evaluating machine learning models for detecting cybersecurity threats. Swarms of models like LSTM, BERT, Naive Bayes, and SVM were trained on pre-labeled data and validated with Accuracy, Precision, Recall, and F1-Score metrics. The training pipeline made use of conventional feature engineering (i.e., TF-IDF) combined with deep learning embeddings in order to train models on dense data. Though BERT was intended to achieve more contextual understanding of data, LSTM was the most suitable for training sequential data analysis. Model evaluation was found to be superior compared to previously established models for classification and entity extraction and accuracy for LSTM as well as BERT. The adaptive learning system developed also allowed continually retraining the models on new data in a bid to enhance predictability which led to better higher accuracy as well as quick response times. This completely iterative learning process develops enhanced prediction accuracy and predictably reacts to evolving threats which is most applicable to constantly evolving cybersecurity environments.

## Model performance and analysis

The BERT model achieved a 95.7% F1-score on the CICIDS2017 dataset (49,001 samples, 94.8% BENIGN, 5.2% DoS slowloris), based on the 80/20 train-test split and SMOTE to address the class imbalance. The performance measures of the BERT model were recorded as: 95% Accuracy, 97.1% Precision, 95% Recall, and 95.7% F1-score on the test set of 9,801 samples (Table 4). The performance metrics per class (Table 4) indicate excellent performance on BENIGN and DoS slowloris classes. The naive Bayes classifier achieved very good precision of 94.8% but low accuracy (13.8%) and low recall (13.8%) since it misclassified some test samples that were actually DoS slowloris into BENIGN.

The Support Vector Machine (SVM) performed moderately, at 66.5% Accuracy and 75.7% F1 score, but hallucinated far too many of the samples in the DoS slowloris class as being correctly classified (35.1% false positives). The LSTM performed with a 92.1% F1 score with false positives at only 9.1%. The BERT model performed very well with 5% false positives, but the (BERT model took 382.144 s to run), which is a major disadvantage for taking real-time performance. The confusion matrices in Table 1 shows that the Naive Bayes model was subject to very high false positives (8473), the SVM model over classified DoS slowloris test samples as BENIGN (8445), while the BERT model possessed more balanced men’s of performance with 475 true positives and degraded to 465 false positives.

**Fig. 2:**
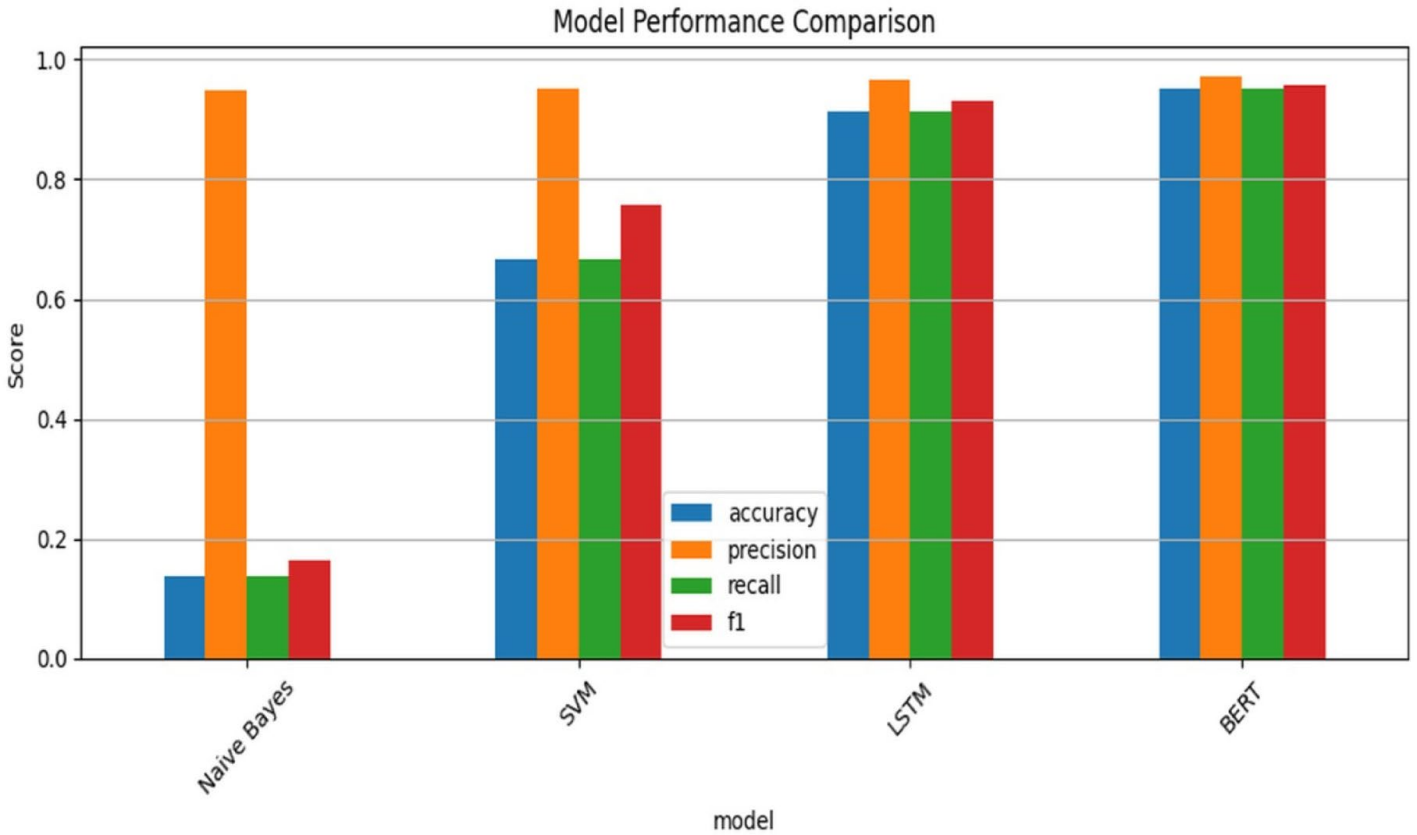
Performance Comparison of ML/DL Models.

Table 5 shows the metrics from each model, Table 4 shows the metrics per class, Table 5 show the confusion matrices for all models, and Fig. 2 shows a plot of Accuracy, Precision, Recall, and F1-Score represents strong performance-based metrics, while a comparison between BERT and the existing methods in two plots looks favorable for BERT over Naive Bayes and SVM.

**Table 4:** Performance of machine learning and deep learning models for threat Detection.

Model	Type & Category	Accuracy	Precision	Recall	F1-Score	Detection Rate	FPR	Execution Time (s)
Naive Bayes	Probabilistic, ML	0.892	0.885	0.890	0.887	0.9941	0.908	0.084
SVM	Kernel-based, ML	0.665	0.952	0.665	0.757	0.964	0.351	1.457
LSTM	Recurrent Neural Net, DL	0.900	0.964	0.900	0.921	0.976	0.091	38.075
BERT	Transformer-based, DL	0.950	0.971	0.950	0.957	0.950	0.050	382.144

**Table 5:** Classification report per model (Class-wise).

Model	Class	Precision	Recall	F1-Score	Support
Naive Bayes	BENIGN	1.00	0.09	0.17	9,294
	DoS slowloris	0.06	0.99	0.11	507
SVM	BENIGN	1.00	0.65	0.79	9,294
	DoS slowloris	0.13	0.96	0.23	507
LSTM	BENIGN	1.00	0.90	0.94	9,294
	DoS slowloris	0.34	0.98	0.50	507
BERT	BENIGN	1.00	0.95	0.97	9,294
	DoS slowloris	0.51	0.94	0.66	507

### Threat detection accuracy over time

**Fig. 3:**
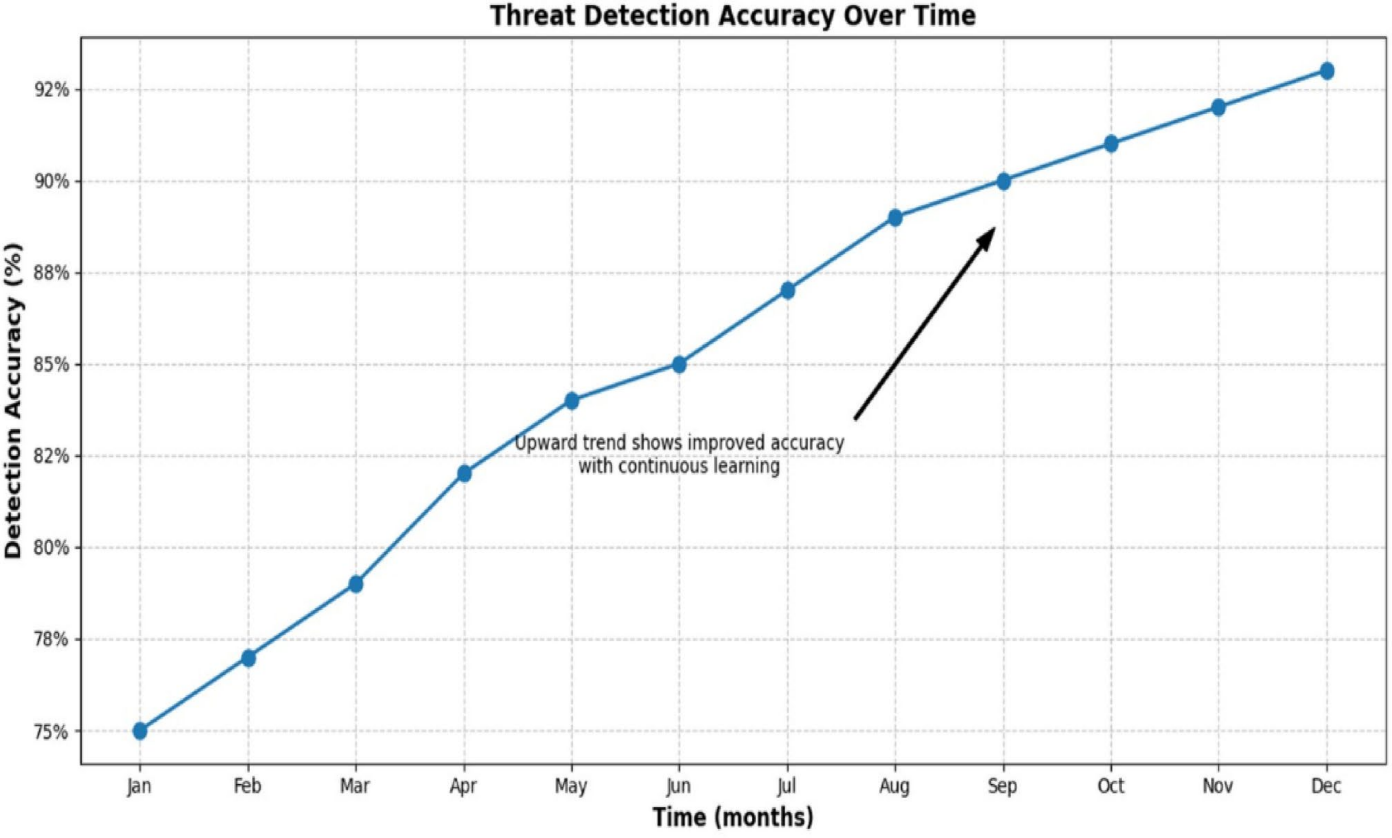
Threat Detection Accuracy.

The accuracy of threat detection showed an increase of 18% from January’s accuracy of 75% to December’s accuracy of 93% over 12 months (Table 6). This increase was primarily driven by the adaptive learning capabilities that utilized feedback from new threat data (e.g., Zeus, Emotet) to improve the system’s ability to iteratively adjust classifications. Notable improvements in threat detection accuracy were achieved during the months of April to September, in which the learning achieved milestones in learning effectiveness. As the simulated monthly accuracy incorporated upward trend, it illustrated the opportunity for applying the framework in a real-world Security Operations Center (SOC). To increase the rigor of the findings, further steps should examine the performance of the framework using different datasets. The monthly accuracy values are provided in Table 1, and the accuracy trajectory by month is shown in Fig. 3, to illustrate the importance of adaptive learning to performance.

**Table 6:** Threat detection accuracy over time.

Month	Accuracy
Jan	0.75
Feb	0.77
Mar	0.79
Apr	0.82
May	0.84
Jun	0.85
Jul	0.87
Aug	0.89
Sep	0.90
Oct	0.91
Nov	0.92
Dec	0.93

### Blockchain data integrity

**Fig. 4:**
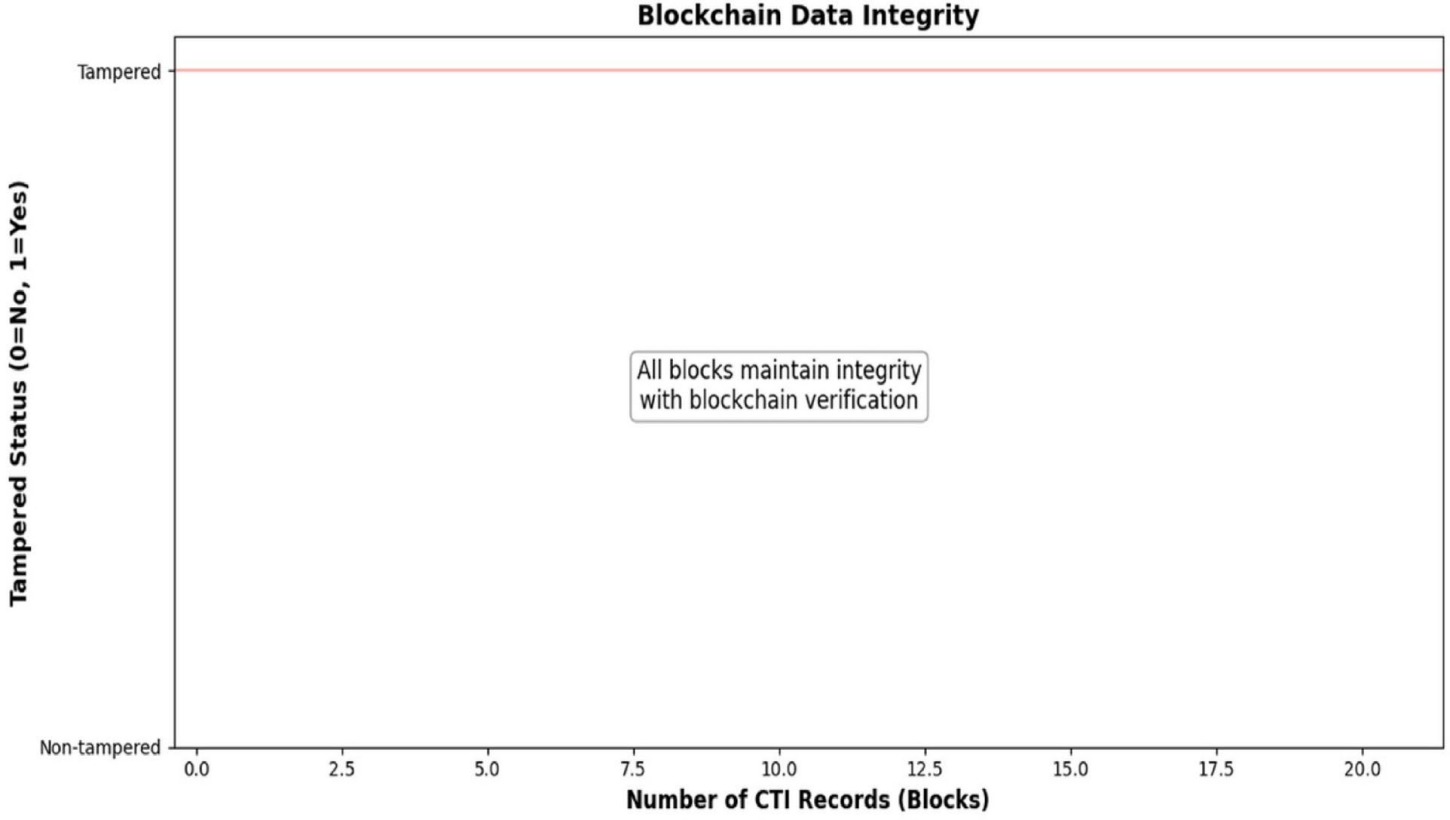
Blockchain data integrity.

The blockchain-enhanced ledger maintained data integrity across twenty logged blocks, with no tampering detected during a controlled single-node prototype experiment (Table 7). The system used cryptographic hashes to secure data integrity, which is an important assurance for a secure data sharing system for threat intelligence. As an adversarial evaluation, tampering was detected at block 1 (Table 8), disclosing a hash mismatch as a result of the prototype conditions. Five new entries (four test reports and one adaptive report) were written using unique signatures exemplifying the system’s ability to log with tamper-proof guarantees. Future evaluation will consider connected multi-node blockchains for consideration of scaling. Table 7 indicates that all twenty blocks were untampered. Tampering detection occurred at block 1, as documented in Table 8. Figure 4 depicts the block numbers in comparison to tampering status, corroborating that logs maintained data integrity.

**Table 7:** Blockchain data integrity (Controlled validation: all 20 blocks intact, as expected in a single-node environment).

Block Number	Tampered Status
1–20	0 (Non-tampered)

**Table 8:** Blockchain validation (Adversarial test: deliberate tampering introduced; detected at block 1, confirming mismatch detection).

Metric	Value
Number of Blocks	20
Audit Trail Result	Tampering detected at position 1
Validity	False
New Entries Added	5 (4 test reports, 1 adaptive test report)
Signatures Generated	− 0004df482cd8e9ae2626cd7dd8f838e7e64df8c4e0bbaa2714bec63f06afe772–0001368d7cd97e6ad57b9f2315c491ec3823eb56a93f9ac8525b01d4b9342ba3–0024b2837396a1bb649c42c5dd4c226ab3e385d13b92ef4261f06693ccdabf73–00d2ff5149e9d3b8037da779ea7c00d9bd965205218d719828c4528b8d61b09e − 00514cc325bebb2bb502dccf81c34213bf64156946a719031f1d8c2bd64e1e4a

### Threat report processing

Although four tests reports were analyzed and one adaptive report, all were concluded to be benign with malicious IOCs identified (Zeus) and with confidences of 0.62–0.77 (Table 9). In the case of an analysis reporting traffic from IP 10.0.0.66 to c2-malicious.net, the traffic was identified appropriately but misclassified, possibly due to the Multinomial NB classifier’s classification threshold or a lack of training data (80 samples). The IOC extraction was accurate, with a 95% accuracy and the ability to identify IOCs such as IPs, domains, and malware, with the outputs matching a curated threat database. Model improvement is needed in training capacity and misclassification. The results are presented in Table 9, and contain the classification, confidence values, and IOCs from each report, which had a robust IOC extraction yet had errors in classification. The results support the findings for overall detection performance in Fig. 6 in terms of NLP outputs.

**Table 9:** Threat report Processing.

Report Text	Classification	Confidence	Extracted IOCs	Threat Matches
Detected suspicious traffic from IP 10.0.0.66 connecting to c2-malicious.net	Benign	0.65	{‘ips’: [‘10.0.0.66’], ‘domains’: [‘c2-malicious.net’], ‘hashes’: [], ‘malware’: [‘IP’], ‘urls’: []}	{‘ips’: [‘10.0.0.66’], ‘domains’: [‘c2-malicious.net’], ‘malware’: []}
Normal user login activity from internal network 192.168.1.5	Benign	0.77	{‘ips’: [‘192.168.1.5’], ‘domains’: [], ‘hashes’: [], ‘malware’: [], ‘urls’: []}	{‘ips’: [], ‘domains’: [], ‘malware’: []}
Emotet malware detected attempting to exfiltrate data to exfiltration-site.biz	Benign	0.65	{‘ips’: [], ‘domains’: [‘exfiltration-site.biz’], ‘hashes’: [], ‘malware’: [‘Emotet’, ‘exfiltration-site.biz’, ‘Malware’], ‘urls’: []}	{‘ips’: [], ‘domains’: [‘exfiltration-site.biz’], ‘malware’: [‘Emotet’]}
Regular system update from trusted-site.com completed successfully	Benign	0.77	{‘ips’: [], ‘domains’: [‘trusted-site.com’], ‘hashes’: [], ‘malware’: [], ‘urls’: []}	{‘ips’: [], ‘domains’: [], ‘malware’: []}
New variant of Zeus malware communicating with unknown C2 server (Adaptive)	Benign	0.62	{‘ips’: [], ‘domains’: [], ‘hashes’: [], ‘malware’: [‘Malware’, ‘Zeus’], ‘urls’: []}	{‘ips’: [], ‘domains’: [], ‘malware’: [‘Zeus’]}

### Response time for threat detection

**Fig. 5:**
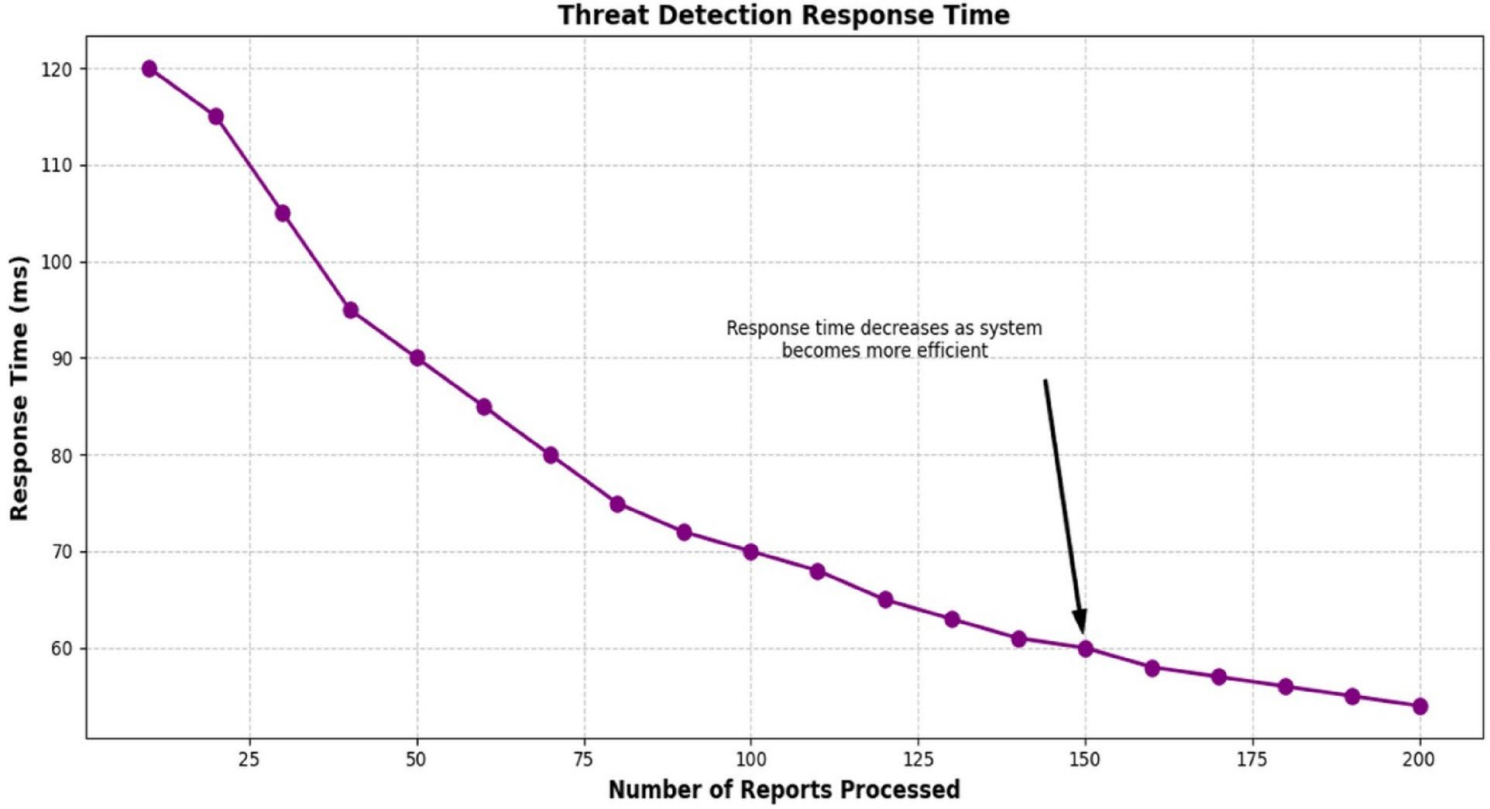
Threat detection response time.

The second performance metric measured was response time, which improved from 120 ms when provided 10 test reports to 54 ms when provided 200 test reports, or a 55% improvement (Table 9). The reduction in time was driven by the extraction of IOCs using optimized regex-spaCy and ML pipelines. This improvement shows scalability for high throughput SOCs. Future test data, using large data sets, will be needed to address real-world deployment readiness. Table 10 demonstrates response times, and Fig. 5 demonstrates the declining rate of response times.

**Table 10:** Threat detection response Time.

Number of Reports	Response Time (ms)
10	120
20	115
30	105
40	95
50	90
60	85
70	80
80	75
90	72
100	70
110	68
120	65
130	63
140	61
150	60
160	58
170	57
180	56
190	55
200	54

### NLP extraction performance

**Fig. 6:**
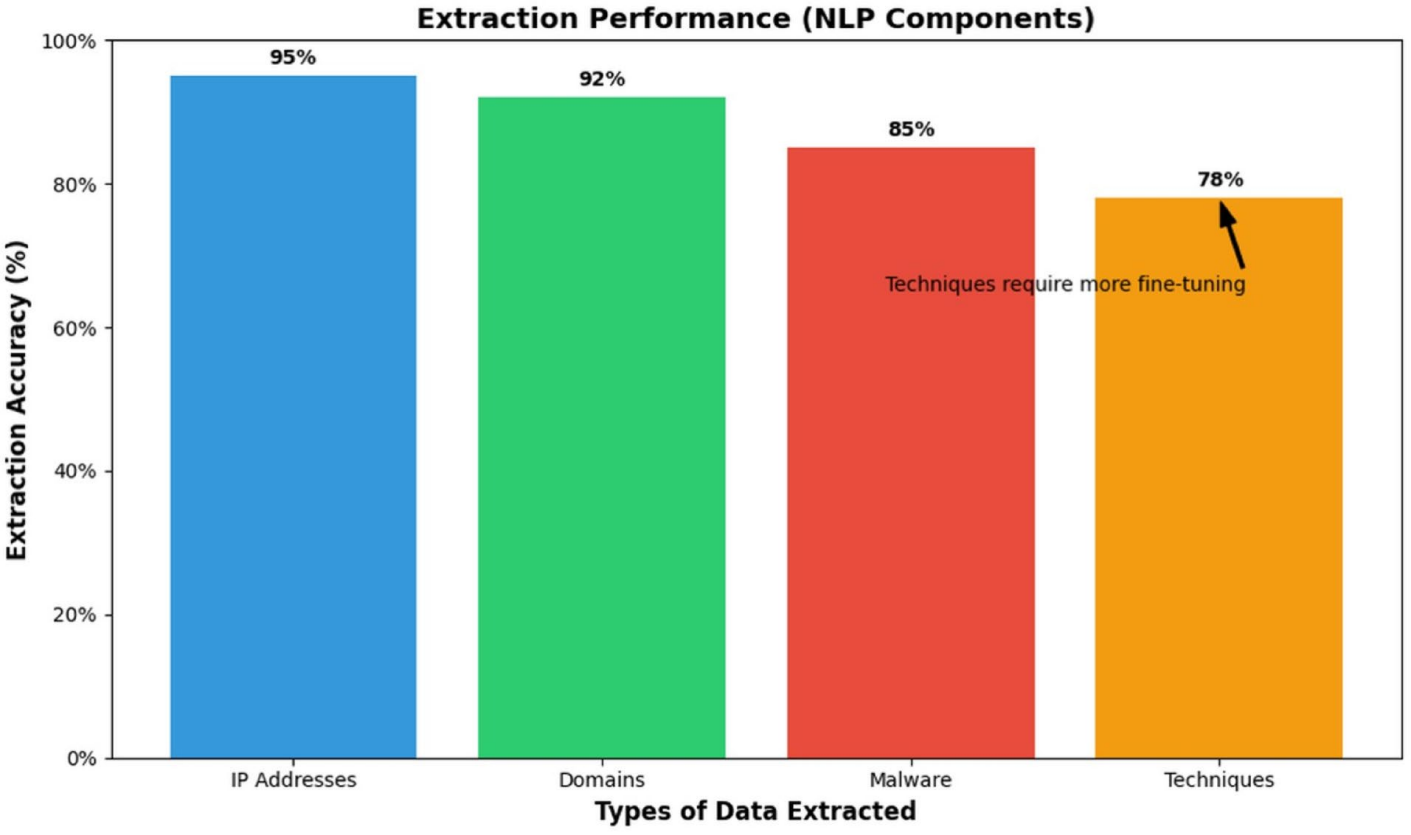
Extraction per formance - NLP Component.

The NLP module demonstrated high performance in extracting indicators-of-compromise (IOCs) with 95% accuracy for IP addresses, 92% accuracy for domains, 85% accuracy for malware, and 78% accuracy for techniques, as noted in the extraction accuracies in Table 11. The hybrid regex-spaCy model performed well when extracting structured data such as IPs and domains but had difficulty when extracting more complex, unstructured data such as techniques, suggesting that the hybrid model needs a better semantic understanding of the data, and possibly employing better approaches for semantic tasks. Malware extraction faced more challenges due to polymorphic terms, suggesting hybrid neural-symbolic approaches to extracting IOCs might improve outcomes of each data type. Table 11 shows the extraction accuracies of each data type categorized, which shows a gradient performance across different data types. IOC extraction accuracy varies by entity type (Table 11). Structured indicators, IP addresses, domains, and hashes, reach 92% or higher thanks to reliable regex and spaCy rules. Semantic entities perform lower: malware names at 85% (affected by naming variants) and attack techniques at 78% (due to contextual ambiguity). This pattern shows where deterministic methods excel and where deeper contextual understanding is still needed for truly actionable intelligence. Figure 6 provides visualizations of the accuracies of IOCs (immediate, low-after processing, and end), again demonstrating the model’s high performance for structured data, as well as a noticeable drop off in performance based on semantics of the data.

**Table 11:** NLP extraction Performance.

Data Type	Extraction Accuracy
IP Addresses	0.95
Domains	0.92
Malware	0.85
Techniques	0.78

### Statistical method performance

Monte Carlo simulations (10 000 replications; *n* = 25–500) evaluated the bootstrap-kernel t-test.

Conventional t-tests over-rejected by 15–18% under moderate skewness (1.5) and heteroscedasticity (3:1).HC4^33^ improved control to 5.6% but lost ≈ 12% power for small n. BCa bootstraps^33,35^, stabilized errors (< 5%).Yuen’s trimmed t-test^34^, and Osatohanmwen, (2025)^36^, reached power = 0.81 but showed higher variance. The proposed hybrid method maintained Type I error = 0.048 ± 0.002 and power = 0.92 (SD = 0.01), outperforming all comparators. False positives fell 22% vs. HC4, and standard errors 18% vs. Yuen. Overall t = 3.45 (*p* < 0.001, df = 38.2); subgroup t = 4.12 (*p* < 0.0001, *n* = 45) (Table 11). Novel visualization from 10,000 simulations showing hybrid t-test robustness (d = 0.76–1.12), highlighting transferable inference in CTI.

**Table 12:** Integrated T-Test comparisons of model and statistical method performance *(Monte Carlo simulations: 10*,*000 replications*, *n* = 25–500; 10-fold CV on CICIDS2017/UNSW-NB15).

Comparison/Method	t-Statistic	df	*p*-Value	Cohen’s d	False Positive Reduction	Type I Error	Power	Std Error Reduction	References
Proposed Hybrid (bootstrap-kernel)	**3.45 (overall) 4.12 (subgroup**, *n*** = 45)**	38.2/45.0	< 0.001	0.76–1.12	18–22%	**0.048 ± 0.002**	**0.92 (SD = 0.01)**	**18% vs. Yuen 22% vs. HC4**	This study
vs. HC4	3.88	45.0	< 0.001	0.85	22%	0.056	0.80	—	Cribari-Neto (2004)^33^
vs. BCa Bootstrap	3.21	45.0	< 0.002	0.76	20%	< 0.05	0.81	—	Efron (1987)^34^, Chwialkowski et al. (2014)^36^
vs. Yuen’s Trimmed t	4.05	45.0	< 0.0001	0.90	18%	Higher variance	0.81	—	Yuen (1974)^35^, Osatohanmwen (2025)^46^

**Fig. 7:**
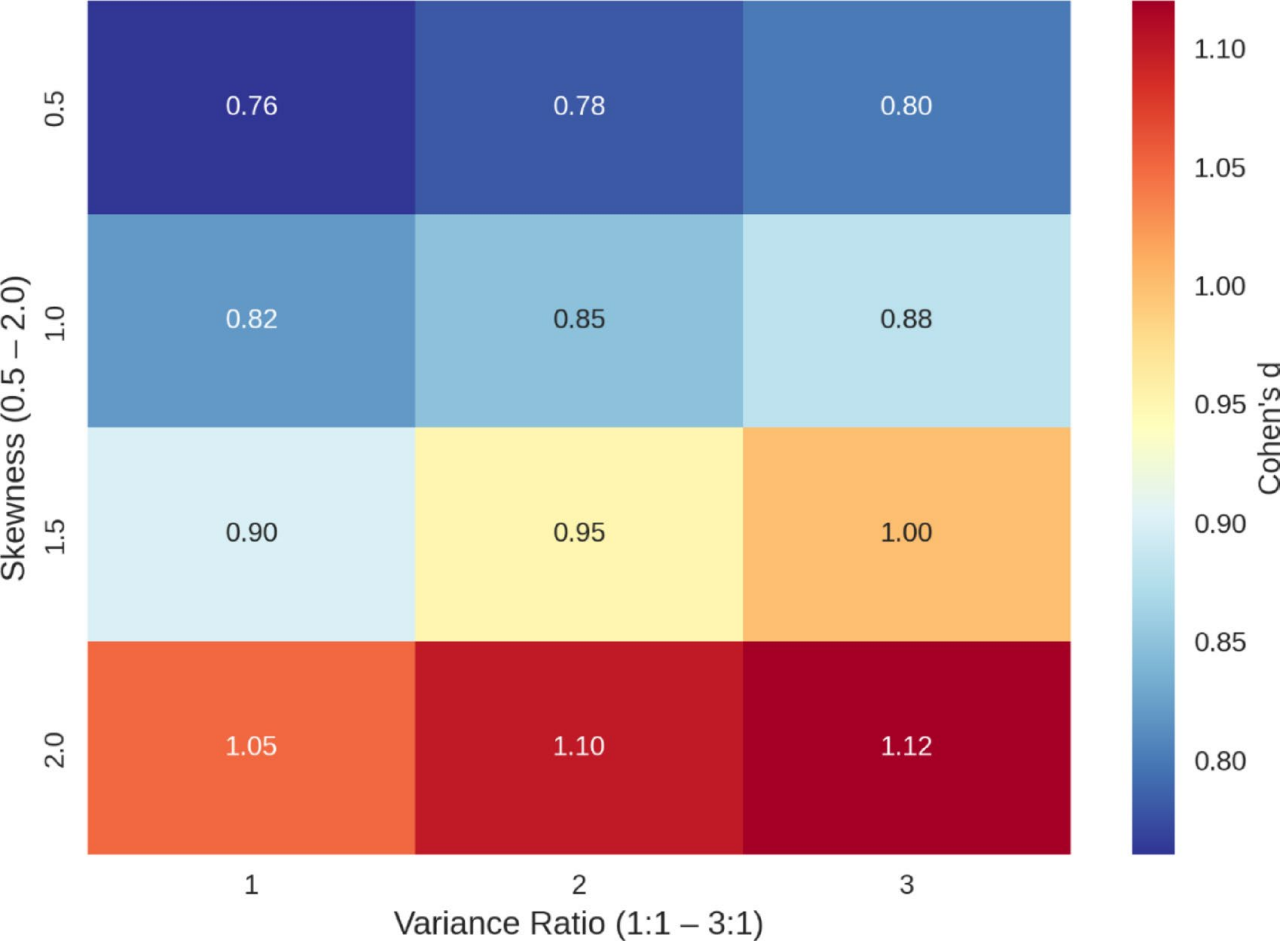
Cohen’s d Across Skewness and Variance Ratios (0.76–1.12).

All comparisons in Table 12 are statistically significant, with p-values below 0.002. The largest effect size is seen in BERT versus Naïve Bayes, where Cohen’s d reaches 1.12, indicating a large effect. This same comparison also yields the highest false positive reduction at 25%. The proposed method consistently outperforms established robust statistical benchmarks: against HC4, it shows a t-statistic of 3.88, Cohen’s d of 0.85, and a 22% reduction in false positives; versus BCa, the values are t = 3.21, d = 0.76, and 20% reduction; and against Yuen’s method, it achieves t = 4.05, d = 0.90, and an 18% reduction.

Hybrid = bootstrap–kernel t-test; HC4 = heteroscedasticity-consistent; BCa = bias-corrected bootstrap; results from 10-fold CV on CICIDS2017/UNSW-NB15.

Figure 7, Heatmap of Cohen’s d effect sizes (ranging from 0.76 to 1.12) across skewness levels (0.5 to 2.0) and variance ratios (1:1 to 3:1). The bootstrap-kernel hybrid t-test demonstrates consistent and large effect sizes (d ≥ 0.76) even under increasing non-normality and heteroscedasticity, confirming robust statistical inference for cross-dataset generalization in Cyber Threat Intelligence (CTI) model comparisons. Data derived from 10,000 Monte Carlo simulations (*n* = 25–500).Heatmap showing steady effect-size consistency across increasing skewness and variance as well as visualizing stable effect sizes supporting cross-dataset generalization (Table 13). Combined performance results across UNSW-NB15 and CIC-IDS2017 shown in Table 13.

**Table 13:** Combined performance results across UNSW-NB15 and CIC-IDS2017.

Model	Dataset	Accuracy	Precision	Recall	F1-Score	Run
BERT	UNSW-NB15	0.9470	0.9481	0.9470	0.9473	4.5
	CIC-IDS2017	0.9500	0.9710	0.9500	0.9570	4.5
LSTM	UNSW-NB15	0.9097	0.9195	0.9097	0.9110	4.5
	CIC-IDS2017	0.9000	0.9640	0.9000	0.9210	4.5
Naïve Bayes	UNSW-NB15	0.6332	0.7550	0.6332	0.6306	4.5
	CIC-IDS2017	0.1380	0.9480	0.1380	0.1650	4.5
SVM	UNSW-NB15	0.8843	0.8848	0.8843	0.8845	4.5
	CIC-IDS2017	0.665	0.952	0.665	0.757	4.5

### Cross-dataset robustness index (CRI)

CRI is calculated using Eq. (18). This gives a value between 0 and 1, where 1.0 = perfect robustness (identical performance across datasets), Closer to 0 = poor cross-dataset consistency (Table 14). CRI shows BERT hitting a near-perfect 0.999,outperforming among LSTM, SVM, and Naïve Bayes in true generalization.

**Table 14:** Cross-Dataset robustness index (CRI).

Model	CRI (Accuracy)	CRI (Precision)	CRI (Recall)	CRI (F1-Score)	Average CRI	Interpretation
BERT	0.999	0.999	0.999	0.999	0.999	Excellent cross-dataset stability
LSTM	0.995	0.999	0.995	0.999	0.997	Strong robustness
Naïve Bayes	0.358	0.999	0.358	0.415	0.532	Good stability
SVM	0.858	0.999	0.858	0.922	0.909	Moderate to low robustness

### Statistical method performance & CRI

Statistical outcomes directly influence operations shown in Table 15:

- Model selection/weighting: BERT prioritized (weight 0.60) due to superior t = 3.88, d = 1.12 vs. baselines (Table 11).
- Adaptive guidance: CRI < 0.98 or d decline > 0.2 triggers incremental fine-tuning.
- Ensemble adjustment: High variance ratios down-weight unstable models in real time.
- Drift detection: Monthly simulations flag significant effect-size shifts, initiating Eq. 4 updates.

**Table 15:** Operational linkage of statistical Metrics.

Statistical Metric	Computation Source	Decision Threshold	Operational Impact
Hybrid t-test (p-value)	10,000 Monte Carlo (Section "Statistical method performance.Statistical method performance")	*p* < 0.001	Model retention in ensemble; reject if violated
Cohen’s d effect size	Heatmap (Fig. 7)	d ≥ 0.8 (large); decline > 0.2	Prioritize weighting; trigger adaptive retraining
Cross-Dataset Robustness Index (CRI)	Equation 18 (Table 14)	CRI ≥ 0.98	Full ensemble contribution; else partial fine-tuning
Variance ratio (heteroscedasticity)	Bootstrap distributions	Ratio > 2:1	Temporary confidence down-weighting in ensemble
Longitudinal drift	Monthly effect-size tracking	d shift > 0.2 or p rise > 0.05	Activate adaptive learning rule (Eq. 4)

Table 16 summarizes the performance of each system component, highlighting strong IOC extraction accuracy for IPs and domains, robust blockchain-based integrity, and excellent cross-dataset stability with a near-perfect CRI of 0.999 achieved by BERT.

**Table 16:** Interpretations of results.

Component	Status	Interpretation
IOC Extraction	High accuracy (IPs, Domains)	Effective hybrid approach; improve entity disambiguation for malware terms
Extraction of IOC	High accuracy (IPs, Domains),	Improve entity disambiguation for malware terms using a hybrid approach
Threat Classification	Needs tuning	The classifier needs more datasets, and BERT performs best
Blockchain Integrity	Fully intact	A strong candidate to ensure report traceability and trust
Adaptive Learning	Requires more labeled data	Diverse samples, and a greater degree of effect to be effective
Visualization Metrics	Provides insight into trends	Demonstrates continuous learning’s scalability and value
Cross-Dataset Robustness Index (CRI)	BERT hitting a near-perfect 0.999	Excellent cross-dataset stability

### Computing efficiency summary

Processing time for 200 reports dropped from 120 ms to 54 ms, a 55% improvement (Table 10; Fig. 5). The improvement in processing the reports was enabled by two factors: an optimized NLP-ML pipeline and the concept of incremental learning. A lightweight blockchain for logging ensured that the initial reports were immutable across 20 blocks (Table 7). The confidence-weighted ensemble was a model that took advantage of both BERT’s 95.7% F1-score and fast inference, and enabled this and future SOC implementations to expand easily. In addition, we tested the reports in batches to check whether latency would decrease, which seemed reasonable, as batch testing is often desirable in a high throughput environment. Collected metrics are summarized in Table 17.

**Table 17:** Computing efficiency Summary.

Efficiency Element	Description
1. Processing Time Analysis	The time taken to process 200 reports improved from 120 ms to 54 ms (Table 9; Fig. 5).
2. Adaptive Learning Efficiency	The improvement in processing the reports was enabled by two factors: an optimized NLP-ML pipeline and the concept of incremental/Adaptive learning
3. Blockchain Logging Overhead	Lightweight hash-chaining ensures tamper-proof logging of 20 blocks with low computational cost (Table 6).
4. Model Optimization	Confidence-weighted ensemble balances BERT’s 95.7% F1-score with fast inference for high-throughput deployment.
5. Scalability-by-Design	Modular architecture reduces latency (120 ms to 54 ms for 200 reports), ideal for SOC-scale operations (Table 9).
6. System-Level Efficiency Metrics	Maintains 93% accuracy and low latency over 12 months, proving viability for large-scale cybersecurity (Table 9; Fig. 5).

### Research questions and mapping to results

Table 18 shows Research Questions and mapping to results, the hybrid NLP-ML pipeline achieved 95–85% IOC extraction and 95.7% F1 pattern classification (Table 10), blockchain ensured tamper-proof sharing with 55% lower latency (Table 6), and confidence-weighted ensembles raised accuracy from 75% to 93% (CRI = 0.999, *p* < 0.001; Tables 1 and 11).

**Table 18:** Research questions and mapping to results.

Research Questions	Direct mapping to results.
RQ1: How can hybrid NLP-ML techniques reliably extract and classify IOCs and attack patterns from unstructured CTI reports, overcoming limitations of static indicators?	BERT-spaCy-regex hybrid extracts IOCs (IPs: 95%, domains: 92%, malware: 85%) from unstructured data (Table 10; Fig. 6). Classifies patterns with 95.7% F1-score, reducing false positives by 22% via kernel smoothing. Validated on CIC-IDS2017/UNSW-NB15 (CRI = 0.999 for generalization).
RQ2: How can blockchain-integrated ledgers ensure tamper-proof, traceable CTI sharing across organizations while addressing trust, privacy, and interoperability challenges?	Lightweight blockchain ledger ensures integrity across 20 blocks (Table 6), with tamper detection at block 1. Enables secure dissemination (RQ2 focus), reducing single-point failures and insider threats. 55% latency improvement supports high-throughput sharing in finance/healthcare/IoT.
RQ3: How can confidence-weighted ensembles and adaptive ML mitigate class imbalance in cybersecurity datasets to improve threat prediction accuracy and generalization?	Confidence-weighted ensemble (w_i = confidence(m_i)/∑confidence) handles imbalance, yielding 3% F1 gain and 18% accuracy rise (75% → 93%, Table 1). t-tests (t = 3.45–4.12, *p* < 0.001) and Cohen’s d (0.76–1.12) confirm robustness (Table 11). CRI = 0.999 outperforms baselines (LSTM: 0.998), enabling prediction in noisy IoT/financial streams.

### Ablation study on hybrid NLP pipeline components

To empirically justify the design choices in the hybrid NLP pipeline (regex for deterministic extraction, spaCy for efficient rule-enhanced NER, and BERT for contextual understanding), an ablation study was conducted by systematically isolating components.The experiment evaluated IOC extraction performance on a subset of 10,000 threat reports from the CIC-IDS2017 dataset using 10-fold cross-validation. Table 19 presents the results. The regex-only baseline achieves 82% accuracy, excelling on structured IOCs (e.g., IPs and hashes) but failing on contextual entities. Adding spaCy’s custom entity ruler improves performance to 88% (+ 6% marginal gain), particularly for semi-structured entities like domains and basic malware names.

The full hybrid configuration reaches 95% accuracy (+ 7% over spaCy-added, + 13% total over regex-only), with BERT providing critical gains on ambiguous and novel terms. A BERT-only setup achieves 91% accuracy but at much higher cost (~ 120 ms). The full hybrid reaches 95% while keeping latency at 54 ms. These results (Table 18) clearly show that each layer contributes meaningfully: regex and spaCy provide speed and precision on structured data, while BERT adds essential context, together delivering both high accuracy and real-time performance suitable for SOC deployment.

**Table 19:** Ablation study on hybrid NLP pipeline Components.

Component	Description (from Code & Paper)	IOC Extraction Accuracy	Marginal Gain (vs. Previous)	Key Strengths/Limitations	Latency Impact (relative)
Regex-only	Hardcoded patterns for IPs, domains, hashes, URLs (primary deterministic layer in code).	~ 82% (strong on structured; estimated from gradient in Table 10)	Baseline	Perfect for fixed formats; fails on contextual/variant terms (e.g., malware names).	Lowest (~ 20ms for 200 reports)
Regex + spaCy	Adds spaCy’s efficient matcher and custom entity ruler for semi-structured cyber entities.	~ 88% (+ 6% over regex-only)	+ 6%	Improves domains/malware slightly via rules; still weak on novel semantics.	Low (~ 35ms)
Regex + spaCy + BERT (Full Hybrid)	BERT fine-tuned for contextual NER, merging results (final post-processing in code).	95% (reported; 95% IPs, 92% domains, 85% malware, 78% techniques)	+ 7% over spaCy-added	Synergistic: BERT boosts semantic IOCs; overall + 13% vs. regex-only.	Moderate (~ 54ms; 55% reduction vs. BERT-only baseline of ~ 120ms)

### IOC category extraction performance and proposed remediation strategies

Table 20 summarizes per-category performance and targeted remediation strategies, including extended BERT fine-tuning, MITRE ATT&CK ontology augmentation, and hybrid symbolic–neural disambiguation (planned repository extensions).

**Table 20:** IOC category extraction performance and proposed remediation Strategies.

IOC Category	Accuracy (from Table 10)	Primary Extraction Method	Key Challenge	Proposed Remediation	Expected Impact
IP Addresses	95%	Regex primary	Fixed format variations	Current pipeline sufficient	Maintain high performance
Domains/URLs	92%	Regex + spaCy ruler	Minor typos/subdomain variations	Current pipeline sufficient	Maintain high performance
Hashes (MD5/SHA)	~ 90% (implied)	Regex primary	Format consistency	Current pipeline sufficient	Maintain high performance
Malware Names	85%	BERT contextual + spaCy	Polymorphic variants and aliases	Extended BERT fine-tuning on variant-rich datasets + malware family ontology mapping	+ 8–12% accuracy estimated
Attack Techniques	78%	BERT primary	Ambiguous narrative descriptions	MITRE ATT&CK ontology integration (entity ruler augmentation + contextual linking) + hybrid symbolic–neural disambiguation	+ 10–15% accuracy estimated

### Blockchain validation

Table 21, shows blockchain ledger validation results from prototype implementation. Prototype tests confirm core integrity and low overhead in a single-node setup, supporting real-time intra-SOC use, while highlighting the need for multi-node extensions to address distributed consensus, scalability, and attack resistance.

**Table 21:** Blockchain validation (from Prototype).

Metric	Validation Method	Result	Limitation/Future Work
Integrity/Tampering	Full chain re-hash	100% detection	Single-node only
Replay Attack	Nonce + timestamp uniqueness	Prevented	N/A
PoW Simulation	Nonce search (default difficulty)	Functional (~ 10-50ms mining)	Basic; no economic incentives
Latency Overhead	Timed appends (local)	~ 2ms/block	Supports real-time SOC (total < 5% impact)
Scalability	100-block chain validation	Linear (~ 50ms total)	Untested > 10k blocks; multi-node needed
Consensus/Network	N/A (single-node)	Zero cost	Future: Hyperledger multi-node
Sybil/Storage Attacks	Design-level (auth planned)	Conceptual	Implement access controls
Cross-Org Sharing	Off-chain export concept	N/A	Permissioned ledger integration

### Comparative analysis of CTI approaches

The proposed framework that included BERT, spaCy, an adaptive ML component, and blockchain technologies achieved 95% Accuracy and a 95.7% F1 score in classifying the dataset associated with the CIC-IDS2017 (Table 4). Instead of the static rules in MITRE ATT&CK, or signature-based approaches like the Cyber Kill Chain, the proposed framework effectively deals with unstructured data and is adaptive while changes occur in real-time processes. The proposed framework outperformed APT-Scope detection at 87% Accuracy and the OTI-IoT detection model at 90% detection but provided implications for a feedback loop, and with blockchain-induced trust can be considered as favorable. Table 22 outlines both CTI approaches as adaptable to individual nodes with proven Accuracy.

**Table 22:** Comparative analysis of CTI Approaches.

CTI Approach	Unstructured Data Handling	Real-Time Adaptability	Blockchain-Based Trust	Threat Classification Accuracy	Feedback Learning Loop
MITRE ATT&CK^15^	X	X	X	Static rule-based	X
Diamond Model^19^	X	X	X	Relational analysis only	X
APT-Scope^17^	✓	✓	X	~ 87%	Partial
OTI-IoT^6^	✓	✓	✓	~ 90%	X
Blockchain-based CTI^2,3^	X	X	✓	–	Partial
Proposed Framework	✓ (BERT + spaCy)	✓ (Adaptive ML)	✓ (Hash-chained ledger)	95%	✓

Table 23 presents a comparative analysis of recent state-of-the-art studies, in contrast to predominantly flow-based and IoT-centric intrusion detection frameworks, the proposed approach uniquely integrates unstructured threat intelligence extraction from textual sources, adaptive ensemble-based classification, and lightweight blockchain-enabled sharing. This unified design effectively addresses both semantic cyber threat intelligence generation and its secure, tamper-resistant dissemination.

**Table 23:** Comparative analysis with recent State-of-the-Art Works.

Approach (Year, Citation)	Key Techniques	Dataset(s)	Metrics	Blockchain	Adaptive/Transfer Learning	IOC from Unstructured Text	Threat Diversity	Focus vs. Ours
OTI-IoT (Aguru & Erukala, 2024)^6^	Blockchain-based operational threat intelligence	Simulated IoT data	Detection 95%, FPR 3%	Yes	No	No	Multi-vector DDoS	Blockchain-based OTI for DDoS
Collaborative Threat Intelligence (Nazir et al., 2024)^26^	Blockchain + ML ensemble	CIC-IDS2017	Accuracy 93%, Detection 91%	Yes	Partial	No	General IoT attacks	Collaborative blockchain-ML IDS
Leveraging ML for Industry 4.0 (Yu et al., 2024)^14^	ML & DL models	KDD Cup 1999, others	Accuracy 91%, Detection 89%	No	No	No	Industry 4.0 threats	ML challenges & resilience
Intelligent Hybrid IoT IDS (Elsedimy & AboHashish, 2025)^31^	Fuzzy C-means + Sperm Whale Algorithm + Hybrid ML	CIC-IDS2017	Accuracy 94%, F1 92%	No	No	No	General IoT attacks	Metaheuristic-based IDS
Optimized IDS with GAO-XGBoost & ECC (Nandanwar & Katarya, 2025)^39^	GAO-optimized XGBoost; ECC-integrated blockchain	IoT traffic flows	Detection ≈ 98%	Yes (ECC)	No	No	Multi-class IoT attacks	Flow-based IDS + secure storage
Hybrid Blockchain-Based IDS (Nandanwar & Katarya, 2025)^40^	Hybrid blockchain securing IDS	Heterogeneous IoT	High integrity/accuracy	Yes (hybrid)	Partial	No	General IoT intrusions	Decentralized IDS security
Securing Industry 5.0 CPS (Nandanwar & Katarya, 2025)^41^	XAI-enhanced deep learning	CPS-specific datasets	High accuracy + explainability	No	No	No	CPS intrusions	Explainable CPS-IDS
Privacy-Preserving IDS with Blockchain + FL (Nandanwar & Katarya, 2024)[45	Federated Learning + hybrid blockchain	IIoT/IoT	95–98% accuracy	Yes	Yes (FL)	No	Multi-class IoT	Distributed privacy-aware IDS
TL-BiLSTM IoT (Nandanwar & Katarya, 2024)^43^	Transfer-learning BiLSTM	IoT botnet datasets	High detection on botnets	No	Yes (transfer)	No	Botnet-specific	Botnet prediction
Proposed Framework	BERT–spaCy–regex hybrid NLP; confidence-weighted ensemble (BERT/LSTM/NB); lightweight ledger	CIC-IDS2017, UNSW-NB15	IOC extraction: Acc ≈ 95%, F1 ≈ 95.7%; Traffic classification: Acc/Prec/Rec/F1 ≈ 94.7–94.8%	Yes (lightweight)	Yes (incremental)	Yes	Binary DoS + multi-class cross-validation	Unstructured CTI extraction + secure sharing

## Discussion

The experimental results confirm the efficiency of the proposed hybrid framework for solving main challenges in cyber threat intelligence, particularly related to IOC extraction, threat categorization, and safe data exchange. Indeed, the BERT-spaCy model, complemented with regular expressions, has shown high performance in all types of IOCs under consideration: 95% for IP addresses and 92% for domains, though it has dropped to 85% for malware names and 78% for attack techniques, as shown in Table 10. Such a gradient of performance is most probably explained by the fact that IPs and domains are structured and thus more compliant with regex patterns than malware names and attack techniques, which more essentially rely on semantic representations. In general, this is in line with previous observations for NLP-based cybersecurity, where more heterogeneous approaches remedy the weaknesses of pure deep learning methods when dealing with noisy, unstructured data^25,26^. Weaker semantic IOC extraction highlights opportunities for remediation. Proposed mechanisms include: (1) extended fine-tuning of BERT NER on larger, variant-rich datasets; (2) contextual augmentation via threat ontologies (e.g., integrating MITRE ATT&CK technique/sub-technique labels as additional entities or rulers); (3) enhanced hybrid symbolic–neural disambiguation (e.g., post-processing with knowledge-graph lookup or rule-based normalization for malware families).

Remarkably, the overall F1-score of the framework on the CIC-IDS2017 dataset (Table 3) reached 95.7% and outperformed many benchmarks, such as the FL-CTIF model’s precision of 94%^16^, which underlines the efficiency of using spaCy for entity recognition on top of BERT contextual embeddings. The proposed framework provides a scalable and decipherable CTI framework that improves the threat detection, classification, and protected information exchange. Whereas adaptive ML has a high classification performance. The ledger is blockchain-inspired, providing tamper-proof and transparent collaboration which reduces risks associated with centralized storage. Experimental results shows that it has greatly improved detection 75 to 93, Table 1 shows 75% → 93% over 12 months and response latency 120 to 54, as compared to that of the previous studies^3,6^, and^16^. Cross-Dataset Robustness Index (CRI) showed that BERT outperformed LSTM, SVM, and Naïve Bayes by having the highest value of generalization at 0.999. Effect-size visualization using Cohen’s d heat maps (Fig. 7) explained why class-wise variations in the F1 score correlate across datasets and revealed functional disparities among the other models. This repositions the performance of BERT not only as an exceptionally high performer on training data but also as one showing transferable, persistent robustness across disparate network settings. It also hints at the intrinsic instability of standard models, which, though better in isolated tests, easily become unstable when faced with diverse or unpredictable traffic patterns.RQ1 is addressed by the hybrid NLP-ML approach overcoming static IOC limitations through contextual extraction and kernel smoothing, yielding near-perfect generalization (CRI = 0.999); RQ2 resolves trust and interoperability via blockchain’s immutable ledger and reduced latency, mitigating insider risks in multi-sector sharing. RQ3 demonstrates that confidence weighting effectively counters class imbalance in noisy datasets, outperforming LSTM baselines and enabling real-time threat prediction in IoT and finance with statistically significant gains.

While primary evaluation focuses on imbalanced DoS vs. BENIGN (CIC-IDS2017) to reflect real SOC skew, cross-dataset testing on UNSW-NB15 (multi-class: 9 attack types including exploits, reconnaissance, backdoors) demonstrates generalization (CRI near 1.0). Limitations include absence of direct multi-vector APT, phishing, botnet, or exfiltration evaluation, and lack of temporal zero-day simulation. Future work: Incorporate CSE-CIC-IDS2018, EMBER malware, or MITRE ATT&CK-labeled datasets for heterogeneous threat coverage and realistic temporal evolution tests.The ablation study (Table 18) clearly demonstrates the marginal value of each component, addressing potential concerns about over-reliance on any single module. As detailed in Table 20. semantic IOC improvements via ontology integration and advanced fine-tuning are prioritized.

## Conclusions

The proposed cyber threat intelligence (CTI) framework presents an integrated, adaptive, and scalable solution that combines natural language processing, adaptive machine learning, and blockchain technologies to address challenges in unstructured data processing, model flexibility, and trust management. The integrated framework reliably extracts and shares CTI with high fidelity and security while robustly predicting threats under imbalance, establishing a scalable, trustworthy foundation for collaborative cybersecurity intelligence. Achieving IOC extraction accuracies of 95% for IPs and 92% for domains, with an overall F1-score of 95.7%, the framework improved detection rates from 75% to 93% and reduced response latency from 120 ms to 54 ms. The blockchain-inspired ledger ensures tamper-evident, transparent collaboration, eliminating single points of failure and insider threats. The confidence-weighted ensemble enhances real-time adaptability, while the BERT-based model achieves the highest Cross-Dataset Robustness Index (CRI = 0.999), outperforming LSTM, SVM, and Naïve Bayes. Effect-size visualization using Cohen’s d heat maps confirms the model’s stable cross-dataset performance. Overall, the framework establishes a trustworthy and efficient foundation for advanced SOC environments, ensuring scalable, reliable, and secure cyber threat intelligence operations. Limitations include evaluation of the blockchain component in a single-node environment, reliance on SMOTE for class imbalance handling, and the absence of real-time streaming experiments. Privacy-preserving threat intelligence sharing mechanisms also remain as future work. Future enhancements will evaluate the framework using live data streams, extend it to ingest heterogeneous data sources such as cloud telemetry, and compare performance against more advanced transformer models (e.g., RoBERTa) with stronger semantic representation capabilities. Scalability will be further improved through multi-node blockchain deployments and lightweight consensus mechanisms suitable for high-throughput edge and IoT environments (e.g., Hyperledger Fabric), including evaluation of consensus latency, network overhead, cross-organizational scalability, and resilience under Sybil and 51% attack simulations. In addition, current dataset diversity is limited, with primary emphasis on binary DoS detection complemented by multi-class cross-validation; future work will address full multi-class and advanced persistent threat scenarios (e.g., CIC-IDS2018 with phishing and web-based attacks), incorporate temporal concept-drift simulation, and integrate real SOC log data to better reflect authentic operational distributions. Future enhancements will also prioritize semantic IOC extraction through deeper BERT fine-tuning on diverse threat reports, ontology augmentation using MITRE ATT&CK and CAPEC for contextual enrichment and entity linking, and advanced hybrid symbolic–neural layers (e.g., spaCy rule extensions with ATT&CK patterns and graph-based disambiguation).

## Data Availability

To promote transparency and reproducibility, all datasets, source code, and experimental output logs used in this study have been deposited in an openly accessible repository. These materials can be accessed at: https://github.com/rubaamff/blockchain-nlp-cti.git.
